# Statistical considerations on real time and extended controlled temperature conditions (ECTC) stability data analysis of vaccines

**DOI:** 10.1016/j.vaccine.2023.08.012

**Published:** 2023-10-06

**Authors:** Deok Ryun Kim, Young Ae You, Hyeon Seon Ahn, Eun Lyeong Park, Jacqueline KyungAh Lim, Katerina Rok Song, Yun Chon, Julia Lynch

**Affiliations:** International Vaccine Institute, Seoul, Republic of Korea

**Keywords:** Extended controlled temperature conditions, Stability, Real-time real conditions, Minimum release potency, Vaccine

## Abstract

**Background:**

Although maintaining vaccines in a strict cold chain has cost and logistical implications in low- and middle-income countries, only a few vaccines have obtained approval for extended controlled temperature conditions (ECTC) application, which permits the administration of vaccines after storage outside of the cold chain for a defined period. We developed a methodology to evaluate stability data and calculate minimum release potency (MRP) in support of ECTC application.

**Methods:**

The methodology is focused on statistical considerations consisting of stability data collection, statistical analysis plan, statistical modelling, and statistical report. It uses mock stability data from a hypothetical product and may serve as a helpful guide for other products. The statistical data analysis is performed using the R program which is an open-source program and validated using the SAS software.

**Results:**

We developed a stability data testing scheme that included 24 lots with six-time points for up to 24 months under real-time and real condition (RT) in the cold chain samples stored at 2–8 °C and 12 lots with six timepoints for 14 days under ECTC samples stored at 40 °C. The log-transformed stability data met the linear regression assumptions and were poolable from representative lots with no significant lot variation. The linear regression analysis model with a common slope and intercept confirmed the stable antigen content over time under RT and ECTC by the mean regression line and 95% confidence interval. Based on the fitted models and the estimated coefficients, the antigen content value of 966 was derived as the MRP under RT for 24 months followed by 14 days under ECTC.

**Conclusion:**

The presented framework of statistical considerations, with practical methods and R program codes to perform statistical analysis, may serve as a guide for developing the CTC data for a vaccine’s stability evaluation prospectively.

## 1 Background

Most vaccines are licensed for storage at 2–8 °C at all points of the supply chain until administration. However, maintaining vaccines in a strict cold chain has cost and logistical implications in low- and middle-income countries. There are only a few vaccines that have obtained approval for extended controlled temperature conditions (ECTC) and administration after exposure for a defined period outside of the cold chain [1], [2].

The World Health Organization (WHO) Guidelines of stability evaluation of vaccines and stability data evaluation of controlled temperature conditions (CTC) is publicly available to support manufacturers to conduct stability data analysis and minimum release potency (MRP) calculation, so that manufacturers can submit the label change request for the vaccine under WHO PQ (Pre-Qualification) requirements [3], [4]. However, some manufacturers may have difficulty following the statistical model in the guidelines to apply to their data due to a lack of technical resources in the company.

The International Vaccine Institute (IVI) has recently worked with collaborators to develop the methodology to evaluate stability data and MRP calculation in support of an ECTC application. Typically, stability testing for ECTC is done with product stored at 40 °C temperature to simulate storage conditions outside the cold chain, while real-time and real-condition stability testing is done on product maintained between 2 and 8 °C (normal fridge temperature).

In an attempt to facilitate more products achieving ECTC, this report is focused on sharing our experience including statistical considerations from the beginning of stability data collection, developing a statistical analysis plan, selection of appropriate statistical models, running statistical programs, and writing a statistical report. It uses mock stability data from a hypothetical product and should serve as a helpful guide for developing the ECTC data for other products. Each step of the statistical analysis, with program source code and outputs, is included as an appendix. The statistical data analysis was performed using the R program (https://www.R-project.org/), which is an open-source program, so that future users can easily mimic the analysis using their stability data.

## 2 Methods

### 2.1 Guidelines for stability data evaluation

There are WHO guidelines and national guidelines (if available) on stability evaluation of vaccines, and International Council for Harmonisation of Technical Requirements for Pharmaceuticals for Human Use (ICH) guidelines of stability data analysis as listed below:

(i) Guidelines on stability evaluation of vaccines. In: WHO Expert Committee on Biological Standardization: fifty-seventh report. Geneva: World Health Organization; 2011: Annex 3 (WHO Technical Report Series, No. 962) [3].
(ii) Guidelines on the stability evaluation of vaccines for use under extended controlled temperature conditions. In: WHO Expert Committee on Biological Standardization: sixty-sixth report. Geneva: World Health Organization; 2016: Annex 5 (WHO Technical Report Series, No. 999) [4].
(iii) Guidelines on stability testing of active pharmaceutical ingredients and finished pharmaceutical products: World Health Organization; 2018: Annex 10 (WHO Technical Report Series, No.1010) [5].
(iv) Guideline on the stability evaluation of vaccines for use under extended controlled temperature conditions (Korean version), 2016, B1-2016-3-009[6].
(v) ICH Guidelines Q1E Evaluation of Stability Data, which contains examples of statistical approaches to stability data analysis [7].

### 2.2 Stability indicating parameters (SIP)

In the guidelines on stability evaluation of vaccines (WHO TRS No. 962, Annex 3), the stability indicating parameters were defined as “parameters that are direct or indirect indicators of vaccine efficacy or safety demonstrated in clinical trials”. They are used to assess product suitability throughout the shelf-life. Determination of these parameters should result in quantitative values with a detectable rate of change. The guidelines intend to discuss vaccine-specific issues and to facilitate the development of “vaccine tailored” stability assessment procedures. It includes the inherent sensitivity of biological substances to changes in environmental conditions, the importance of tests reflecting potency, and their degree of uncertainty. A key stability indicating parameter for a vaccine has to be identified and a stability profile has to be established. Robust assays characterized by low experimental variability may be preferred to assay suffering large variability. While assay variability can somewhat be offset by increasing the number of samples tested (see below), selecting a robust assay in the first place may pre-empt that.

Most vaccines consider potency as a stability indicating parameter (SIP) that reflects the potential impact of real-time and real-condition and extended controlled temperature conditions on the antigenicity and subsequent efficacy of a vaccine (WHO TRS No. 962, Annex 3). However, various other stability parameters should be evaluated and considered such as antigen content, appearance, pH, general safety, specific toxicity, antimicrobial agent content, completeness of absorption, sterility, adjuvant (absorbent) content and changes in physicochemical properties. It is likely that, during the consultation with regulators, product developers will be asked to provide the data upon which the SIP was selected, and other parameters rejected, indicating that selection of the SIP is an important foundation for the analysis.

### 2.3 Stability data collection plan

Stability data evaluation should plan to include enough number of test lots, test time points, and test replicates to mitigate statistical uncertainty and fit the adequate statistical analysis model.

#### 2.3.1 Number of test lots

As per guidelines, at least 3 lots are required for stability data evaluation. However, because of inherent assay and manufacturing variability, it may be necessary to increase the number of lots to mitigate the statistical uncertainties of point estimates. However, there may be a possibility of additional variability introduced when adding lots manufactured at different time points.

#### 2.3.2 Number of test time points

As per guidelines (WHO TRS No. 962), the testing frequency at the real-time and real-condition (RT) in the normal cold chain (2–8 °C) should be every 3 months over the first year, every 6 months over the second year, and annually thereafter throughout the proposed shelf-life expiration date. In the case of accelerated storage conditions, a minimum of three testing time points, including the initial and end timepoints, for example, 0, 3, and 6 months, are recommended. In the case of a 24-month shelf-life, the testing time points should be at least 0, 3, 6, 9, 12, 18, and 24 months under real-time and real-condition, and three-time points under ECTC. As the testing frequency mentioned may not be applied to all vaccines, the stability data collection plan needs to consider the characteristics of the vaccine and to have consultation with regulators the same as SIP selection.

#### 2.3.3 Number of test replicates

The number of assays to be performed to support RT and ECTC can be quite large and the cost high. In order to reduce costs, it may be to use only duplicates (rather than the recommended triplicates) for each sample [8], [9]. However, it may be useful to consider a higher number of replicates due to the high variability of potency assays. For example, if there is high variability (e.g., Coefficient of variation > 20% cut-off) between two test replicates in a dual well set-up, we may lose both datapoints because it will not always be clear which of the replicates is an outlier, or we may keep both datapoints to maintain sufficient data points and number of lots;, whereas if more than two replicates are performed we may identify an outlying data-point and eliminate the outlier from the test replicates and thereby improving the analysis.

Overall, it is important to plan stability data collection prospectively, including the selection of lots, to reduce the degree of uncertainty as much as possible in the point estimate of a parameter in the statistical model, and to include time points for testing each lot to adequately cover the requirements. Further, if assay variability is a concern, one should consider testing an adequate number of replicates per sample to ensure all data points can be included in the analysis.

The following Table 1 is an example of a mock stability testing scheme for both RT and ECTC, which starts at 6 months intervals for 2 years. The first test set includes the latest exposure month 24 for ECTC stability data collection. Different batches for each test set B, C, and D are included for RT and ECTC stability tests. The same batches as used in the RT stability test are also used for ECTC testing. Depending on the lot variation over time, an increased number of batches needs to be considered.

**Table 1:** Stability data testing schema.

Test set	Stability testing method	Number of Batches	Manufacture Date	Testing timepoints	Test Completion Date
A	RT	3 Batches	Q2Y1	Month 0, 3, 6, 9, 12, 18, 24	Q2Y3
	ECTC	3 Batches	Q2Y1	Day 0, 3, 7, 10, 12, 14at each exposed Month 0, 6, 12, 24	Q2Y3
B	RT	3 Batches	Q4Y1	Month 0, 3, 6, 9, 12, 18, 24	Q4Y3
	ECTC	3 Batches	Q4Y1	Day 0, 3, 7, 10, 12, 14at each exposed Month 0, 6, 12, 24	Q4Y3
C	RT	3 Batches	Q2Y2	Month 0, 3, 6, 9, 12 18, 24	Q2Y4
	ECTC	3 Batches	Q2Y2	Day 0, 3, 7, 10, 12, 14at each exposed Month 0, 6, 12, 24	Q2Y4
D	RT	3 Batches	Q4Y2	Month 0, 3, 6, 9, 12 18, 24	Q4Y4
	ECTC	3 Batches	Q4Y2	Day 0, 3, 7, 10, 12, 14at each exposed Month 0, 6, 12, 24	Q4Y4

[Note] RT: Real-time, Real conditions in the normal cold chain (2–8 °C); ECTC: Accelerated storage conditions (40 °C); Q: Quarter; Y: Year.

### 2.4 Statistical analysis plan development

The statistical analysis plan (SAP) is developed to describe details of the statistical methodology and analyses to be performed as outlined in the guidelines titled “Guideline on the stability evaluation of vaccines for use under extended controlled temperature conditions” published by WHO (2015) as well as national regulatory guidelines available, and the scope of the SAP and who will perform the analysis.

**Fig. 1:**
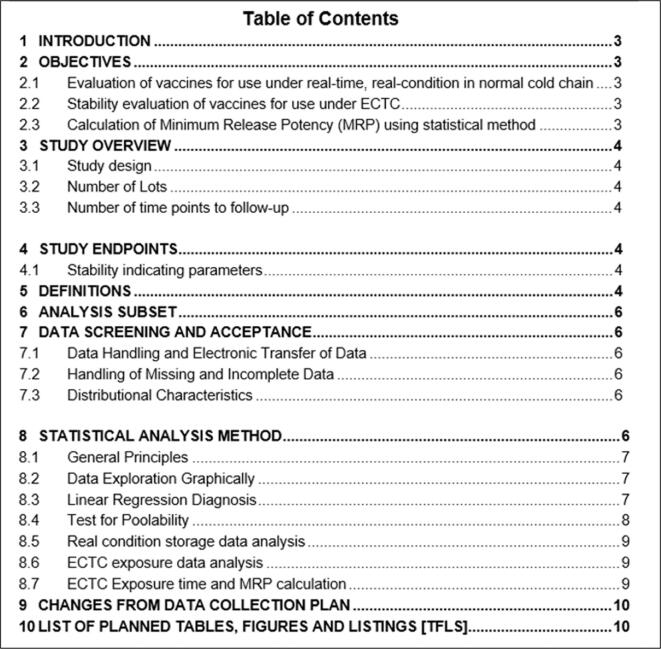
Contents of Statistical Analysis Plan.

This paper provides the SAP template (Supplementary Data A). Fig. 1 presents the contents to be considered in the SAP development.

### 2.5 Consideration of statistical models

As per guidelines, an appropriate statistical model such as regression analysis should be considered for both RT of long-term and ECTC stability evaluation and MRP calculation. However, the standard statistical models often assume that data are normally distributed with constant variance, not depending on the mean of the data. Data that violates these assumptions can be in line with the assumptions by the application of a transformation. In the mock data we use for this example, we have variability that changes with the mean in a non-linear fashion. After checking several transformation candidates (e.g., log, square root, etc.) by the mean-range plot (the range is plotted against the means for each time point) of the untransformed data, the log transformation is selected for variance stabilization.

The following linear regression assumptions are verified for the log-transformed data: linear relationship, influence, normality of residuals, homogeneity of variance of the residuals, and independence.

#### 2.5.1 Nonlinearity

The relationship between the predictor (time in months) and the log-transformed antigen content should be linear. This can be evaluated through observation of the scatter-plot between the outcome measurements and predictor (time) to see if the nonlinearity is present by adding both linear line and LOESS (Locally Weighted Smoothing) line, to see the relationship between variables, and to observe trends.

#### 2.5.2 Unusual and influence data

There are three ways that an observation can be unusual: outlier (is an observation with large residual), leverage (an observation with extreme value on a predictor variable which affects the estimate of regression coefficient), and influence (influential if removing the observation substantially changes the estimate of coefficients; this can be thought of as the product of leverage and outlier-ness).

In the R program, the olsrr package [10] offers the following tools to detect influential observations with threshold: Studentized Residual Plot, Cook’s D Bar Plot, Studentized Residuals vs Leverage Plot, DFFITs Plot.

Influential outliers are of the greatest concern. They should never be disregarded. Careful scrutiny of the original data may reveal an error in data entry that can be corrected. If they remain excluded from the final fitted model, they must be noted in the final report or paper. Supplementary Data C provides R program codes.

#### 2.5.3 Normality of residuals

In the Shapiro-Wilk W test for normality, the p-value is based on the assumption that the distribution is normal.

#### 2.5.4 Heteroscedasticity

One of the main assumptions for the ordinary least squares regression is the homogeneity of variance of the residuals. If the variance of the residuals is a non-constant, then the residual variance is said to be heteroscedastic. There are graphical methods [(plot the residuals versus fitted (predicted) values]) and non-graphical methods (Breusch-Pagan test and White test).

#### 2.5.5 Independence

Since the data will be collected on the same lots over time, the assumption of independence can be broken. In this situation, the errors for observations between adjacent time points will be more highly correlated than for observations more separated in time. Durbin-Watson’s d tests the null hypothesis that the residuals are not linearly auto-correlated. As a rule of thumb values of 1.5 < d < 2.5 show that there is not auto- correlation.

#### 2.5.6 Poolability

Prior to poolability test, the normality of outcome measurements will be checked. Upon normality test and reducing the effect of outliers in the linear regression model, all outcome measurements will be natural logarithmically transformed for analysis.

According to the following criteria (Ref. ICH Q1E. Evaluation for Stability Data. B3.2. Tests for Poolability), the test of equality of slope and test of intercept will determine if the combined data will be used in the model Table 2.

**Table 2:** Criteria for poolability.

Equality of Slope p-value	Equality of Intercept p-value	Final Reduced Model		
		Slope	Intercept	Description
≤ 0.25	NA	Separate	Separate	Not Parallel
> 0.25	≤ 0.25	Common	Separate	Parallel
> 0.25	> 0.25	Common	Common	One Line

### 2.6 Stability evaluation and MRP calculation

Based on the final model, the mean regression line and 95% confidence intervals are estimated. If the mean regression line and lower limit of the 95% confidence interval over time are above the minimum potency of antigen content, the stability is satisfied.

The coefficients of intercept and slope of the final model are used in the formula of MRP calculation. In the case of the negative slope, the zero (0) value is used in the calculation, conservatively.

## 3 Result

### 3.1 Reporting statistical analysis results

The statistical analysis should be performed according to the planned statistical analysis. The statistical analysis reports consist of stability data collection plan, analysis of stability data obtained under real-time and real-condition and extended controlled temperature conditions, and the minimum release potency calculation using the given shelf-life and the rate of decay of the potency over both the long-term storage temperature and the extended controlled temperature conditions to demonstrate that the antigen is above the lower limit of potency. The stability evaluation results will be presented in tabular formats and graphical formats for antigen contents over time for each batch/lot. This paper provides the Statistical Analysis Report Template (Supplementary Data B) to be considered when summarizing the analysis results.

### 3.2 Stability data collection and descriptive summary

The planned stability data collection was presented in Supplementary Data A. The stability data time points of RT and ECTC were presented in Table 3.

**Table 3:** Distribution of real-time stability data time points by the number of lots.

RT	Data time points (Months) for real-time stability							
	M0	M3	M6	M9	M12	M18	M24	Total
24 lots	24	12	24	12	24	12	15	123

ECTC	Exposed Month	Data time points (Days) for ECTC stability						

D0	D3	D7	D10	D12	D14	Total

12 lots	M0	12	12	12	12	12	12	72
12 lots	M6	12	12	12	12	12	12	72
12 lots	M12	12	12	12	12	12	12	72
3 lots	M24	3	3	3	3	3	3	18

[Note] RT: Real-time Real conditions in the normal cold chain (2–8 °C); ECTC: Accelerated storage conditions (40 °C); M: Month; D: Day.

**Fig. 2:**
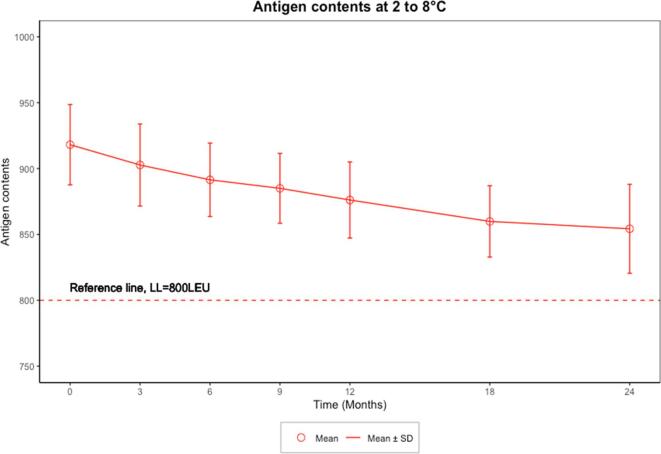
Mean and SD of antigen contents under RT data over time.

**Fig. 3:**
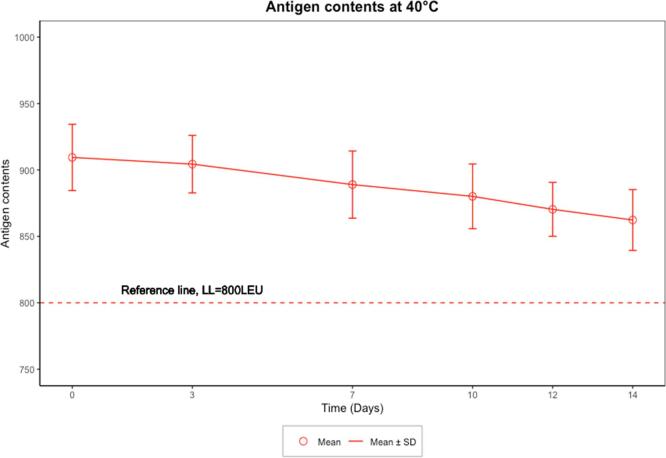
Mean and SD of antigen contents under ECTC data over time.

Fig. 2, Fig. 3 graphically presented the mean and standard deviation (SD) of observed contents, which were stable and above acceptable release criteria at each time point for 24 months under RT and 14 days under ECTC.

### 3.3 Statistical analysis modelling

An appropriate statistical model should be considered for both RT of long-term and ECTC stability evaluation and MRP calculation. The log-transformed antigen content was used for checking the following linear regression assumptions: linear relationship, influence, normality of residuals, homogeneity of variance of the residuals, and independence. This section provides an R program code and helps how to interpret the outputs.

#### 3.3.1 Nonlinearity

**Fig. 4:**
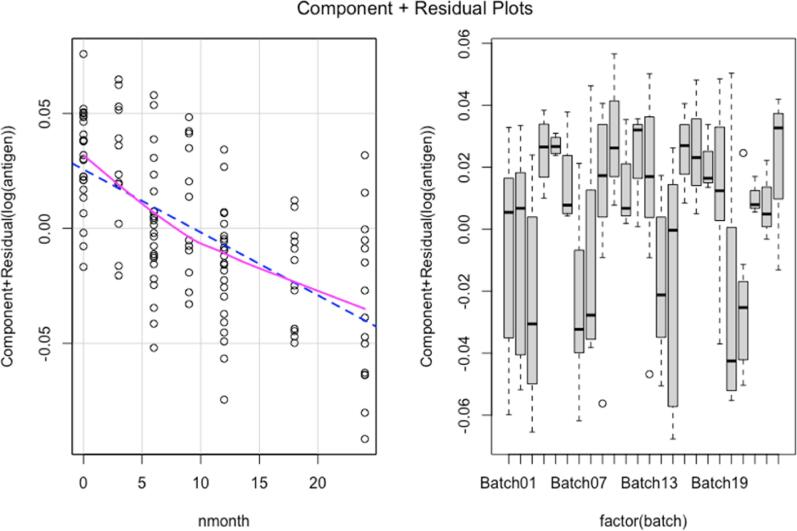
Nonlinearity diagnosis.

The crPlots() function was used for visually seeing whether predictors have a linear relationship with the dependent variable. crPlots() shows Component + Residual (Partial Residual) Plots for linear and generalized linear models, along with a smoothing line that models the residuals of the predictor against the dependent variable (i.e., the loess line). The dashed line in Fig. 4 represents the line of best fit. If the smoothing line seems to be similarly linear as the dashed line, we have adequate linearity; if the smoothing line appears curved relative to the dashed line, we likely have a linearity problem. Here we can control the parameter α which controls the degree of smoothing. For α < 1, the neighborhood includes proportion α of the points, and these have tricube weighting. For α > 1, all points are used, with the ‘maximum distance’.

#### 3.3.2 Unusual and influence data

**Fig. 5:**
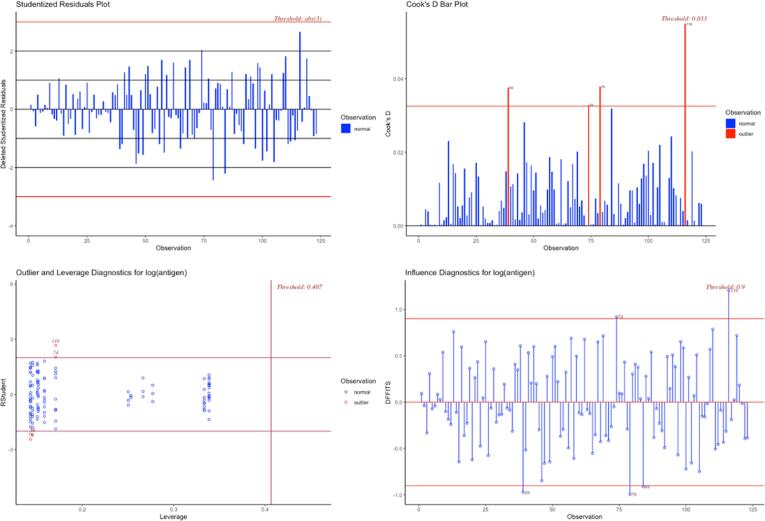
Unusual and influential data checking.

The plots such as studentized residual plot, Cook’s D bar plot, studentized residuals versus leverage plot, DFFITs plot in the olsrr package [10] were used for detecting influential observations with the threshold Fig. 5.

Influential outliers are of the greatest concern and therefore should never be disregarded. A careful review of the original data may reveal an error in data entry that can be corrected. If they remain excluded from the final fitted model, they must be noted in the final report or paper. The R program codes can be found in Supplementary Data C.

#### 3.3.3 Normality of residuals

The shapiro_test() function was used for normality check based on the value of the test statistic and its p-value. The null hypothesis of the test is that “sample distribution is normal”. If the test is significant, the distribution is non-normal. In the case of non-normal residuals, we may consider the non-linear (e.g., polynomial or quadratic) transformation of independent variables. The log-transformed stability data met the normality assumption (p = 0.896 for RT and p = 0.315 for ECTC data).

#### 3.3.4 Heteroscedasticity

The bptest() function, Breusch-Pagan was the test for heteroscedasticity. The bptest() tests whether the variance of errors from regression is dependent on the values of an independent variable. The White Test, to see whether the multiplicative relationship exists, was performed by adding an indicator variable such as the I(x^2) term that includes a polynomial squared term to the regular Breusch-Pagan test. The difference between the BP test and White Test is the existence of multiplicative terms and squared terms in the auxiliary regression. In the case of the heteroskedasticity of error term of the model, the models (e.g., autoregressive conditional heteroscedasticity model) allowing non-constant variance can be considered. There was no evidence of heteroscedasticity on the stability data from both tests (p = 0. 1964 and 0. 4299 for each test for RT data, p = 0. 1622 and 0.2841 for each test for ECTC data,).

In addition, a plot of residuals vs fitted values is shown in Supplementary Data C (section 4.5.3) to visually check the shape of relationship. If the model does not satisfy the linear model assumption, one will find residuals take on a defined shape or a distinctive pattern. The red line through the scatterplot should also be straight and horizontal, not curved if the linearity assumption is satisfied. To assess the homoscedasticity assumption, please make sure that the residuals are equally spread around the y = 0 line.

#### 3.3.5 Independence

The dwtest() function was used to perform the Durbin-Watson test for autocorrelation of disturbances. The null hypothesis of the test is no correlation among the residuals, and the alternative hypothesis is that the residuals are autocorrelated. In the case of the violations of independence (e.g., serial correlation by time lag) of error term of the model, the models (e.g., random effect model or autoregressive model) allowing auto-correlated variance can be considered. There was no evidence of autocorrelation on the stability data from both tests (p = 0.3106 for RT and p = 0.7821 for ECTC data).

#### 3.3.6 Poolability

The Poolability of the different batches data was checked using the full model included variables of time and batche as main effect term and interaction term of time and batch. The reduced model included main effect terms only. In the example using R code,

Full model:

lm(log(Ogawa) ∼ nmonth + factor(batch) + nmonth:factor(batch), data)

Reduced model 1 (non-interaction term):

lm(log(Ogawa) ∼ nmonth + factor(batch), data)

Reduced model 2 (no batch variable):

lm(log(Ogawa) ∼ nmonth, data)

The poolability tests of the different batches data confirmed the model including common intercept and common slope (p > 0.25) of time variable with no significant batch-to-batch variation. The combining (pooling) of data from all batches was acceptable and the estimates derived from the overall linear regression model (Reduced model 2 in the example above) were used in the MRP calculation. In case of the poolability tests of the different batches data confirmed significant (p < 0.25) variation among batches, the model includes batch variable as the separate slopes to account for batch variations. With the evidence for different slopes among the batches, the estimates derived from the model by controlling batch variation alone (Reduced model 1 in the example above) or by controlling batch variation as well as interaction term (Full model in the example above).

### 3.4 Evaluation of vaccines for use under real-time, real condition in normal cold chain

**Fig. 6:**
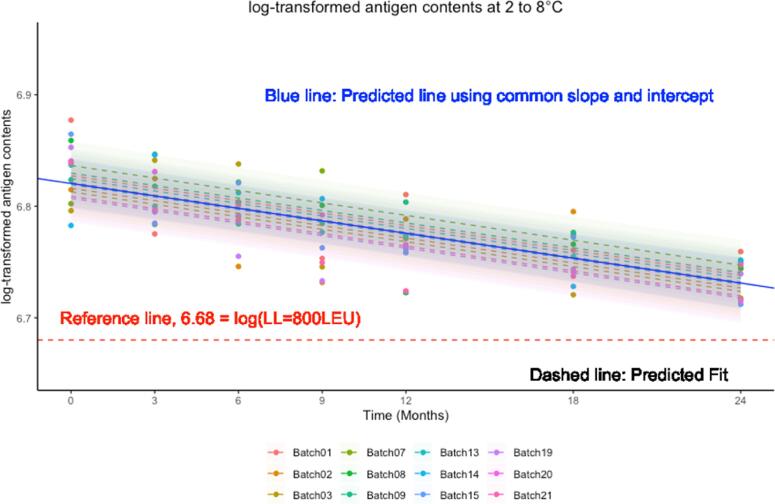
Antigen content trend under RT over time.

Based on the final model, the mean regression line and 95% confidence intervals were estimated. If the mean regression line and lower limit of the 95% confidence interval over the time are above the minimum potency of antigen content, the stability is satisfied. Fig. 6 presents the observed antigen content in the mock data set and predicted mean regression linear lines with its 95% confidence intervals. It showed that all observed antigen contents and the lower bound of 95% confidence intervals were above acceptable release criteria over the time. It showed stable antigen content over time within batches but had variable antigen content at 0 month and different variability over time by lot.

### 3.5 Stability evaluation of vaccines for use under ECTC

**Fig. 7:**
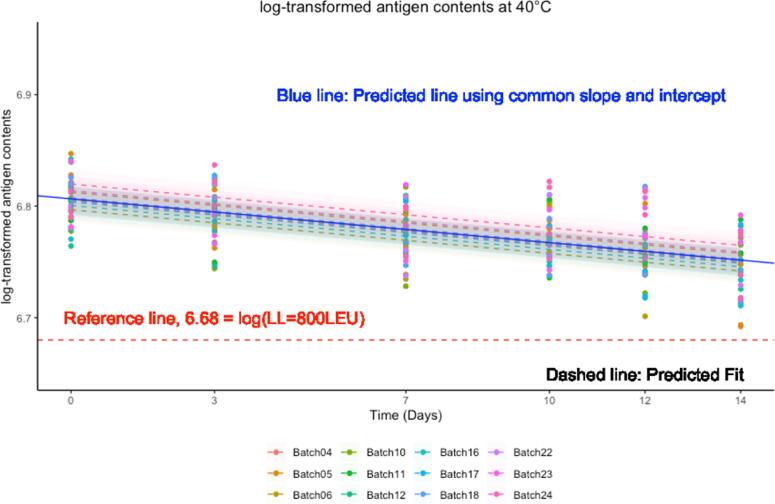
Antigen content trend under ECTC over time.

Based on the final model, the mean regression line and 95% confidence intervals were estimated. If the mean regression line and lower limit of the 95% confidence interval over the time are above the minimum potency of antigen content, the stability is satisfied. Fig. 7 presents the observed antigen contents and predicted mean regression linear lines with its 95% confidence intervals. It showed that all observed antigen contents and the lower bound of 95% confidence intervals were above acceptable release criteria over the time. It showed stable antigen content over time within batches except a few data points at days 12 and 14 but had variable antigen contents at 0 month and different variability over time by batch. The estimated mean regression lines showed a small negative value of slope.

### 3.6 ECTC exposure time and MRP calculation

The coefficients of intercept and slope of the final model from the models of stability data under RT and ECTC was used in the formula of MRP calculation. In the case of the negative slope, the zero (0) value was used in the calculation, conservatively. In the MRP calculation, we considered several scenarios such as 24 months of shelf-life under RT, 3, 14 days of ECTC exposure duration, and 0, 6, 12, and 24 months of RT conditioning storage before ECTC exposure. Table 4 provides the estimated coefficients when the model uses 0–24 M data set under RT condition. The estimates derived from the models were used in the MRP calculation formula. Table 5 presents the estimated coefficients when the model used the pooled 12 lots (D0-D14) data set under ECTC and the calculated MRP from both RT and ECTC models.

**Table 4:** Statistical analysis of antigen contents under RT.

**Data set** **(unit: months)**	**Shelf-life (months)**	**Pooled slope** **(per month)**	**SE of slope**	**Residual STD**	**Uncertainty**
24 lots (0–24 M)	24	−0.003723	0.000377	0.0271	0.0469

**Table 5:** Statistical analysis of antigen content under ECTC.

**Data set** **(unit: days)**	**Days at ECTC**	**Pooled slope (per months)**	**SE of slope**	**Residual STD**	Combined uncertainty *	**MRP**
Pooled 12 lots (D0-D14)	3	−0.117,480	0.009,942	0.0250	0.0456	924
Pooled 12 lots (D0-D14)	14	−0.117,480	0.009,942	0.0250	0.0462	966
Pooled 12 lots (D0-D14) exposed at M0	3	−0.118,629	0.018,841	0.0263	0.0457	925
Pooled 12 lots (D0-D14) exposed at M0	14	−0.118,629	0.018,841	0.0263	0.0479	968
Pooled 12 lots (D0-D14) exposed at M6	3	−0.126,544	0.018,649	0.0260	0.0457	925
Pooled 12 lots (D0-D14) exposed at M6	14	−0.126,544	0.018,649	0.0260	0.0478	971
Pooled 12 lots (D0-D14) exposed at M12	3	−0.102,528	0.017,323	0.0242	0.0470	923
Pooled 12 lots (D0-D14) exposed at M12	14	−0.102,528	0.017,323	0.0242	0.0488	960
Pooled 3 lots (D0-D14) exposed at M24	3	−0.136,440	0.034,428	0.0240	0.0460	926
Pooled 3 lots (D0-D14) exposed at M24	14	−0.136,440	0.034,428	0.0240	0.0527	981

[Note] * Uncertainty at RT for 24 months followed by 3 and 14 days at ECTC; M: Month; D: Day.

For MRP calculation the following formula was used and the estimated coefficients from Table 4, Table 5 were used. For example, the potency value, 966, was derived as the MRP under RT for 24 months followed by 14 days under ECTC.

**Table:** 

**MRP** = LL – antigen content at the end of storage (RT plus ECTC),
[on natural log-transformed data]
= exp[log(LL**)** – (t_2_8_ ∙ b_2_8_ + t_ECTC_ ∙ b_ECTC_ – U)]
MRP = exp[log(8 0 0) – (24*(−0.003723) +
(14/30)*(−0.117480) – 0.0462)] = 966
where,
t_2_8_ is time in months at 2–8 °C,
b_2_8_ is decay slope at temperature 2–8 °C,
t_ECTC_ is time in months at 40 °C,
b_ECTC_ decay slope at temperature 40 °C,
U is combined uncertainty at 2–8 °C and uncertainty followed by exposure days at 40 °C

The results of the other scenarios and the interpretation of stability evaluation with R program codes and outputs are provided in Supplementary Data C and the sample mock stability data in Supplementary Data D.

## 4 Conclusion

The stability of mock data of product batches during long-term storage for up to 24 months under cold chain conditions and short-term storage for up to 14 days under extended controlled temperature conditions was confirmed by the result of evaluating graphical visualization and lower bound of the estimated mean regression line. A 24-month shelf-life at RT was determined to be appropriate and 14 days at ECTC prior to immunization was considered acceptable. Different specifications for release should be established for antigen contents for the product. The product batches which contain 923 LEU at release and are stored for 12 months at RT followed by an exposure of 3 days at ECTC, were expected to contain above 800 LEU (LL). Alternatively, the release specification of 981 LEU might be tightened to allow for 14 days longer than 3 days of ECTC exposure.

This paper was to summarizes the experience gained while analyzing the RT and ECTC data for antigen content using mock stability data to provide a framework for discussing statistical considerations for evaluation of other vaccine products with practical methods and program codes on the R program to perform statistical analysis. It may serve as a guide for developing the CTC data for other vaccine’s stability evaluation prospectively.

## 5 Availability of supporting data

This paper describes a mock stability data collection plan and statistical analysis result of mock stability data. The source code of the statistical stability data analysis is made available for public use.

## CRediT authorship contribution statement

**Deok Ryun Kim:** Conceptualization, Investigation, Formal analysis, Supervision, Writing (original draft). **Young Ae You:** Formal analysis, Writing (review & editing). **Hyeon Seon Ahn:** Formal analysis, Writing (review & editing). **Eun Lyeong Park:** Formal analysis, Writing (review & editing). **Jacqueline KyungAh Lim:** Investigation, Writing (review & editing). **Katerina Rok Song:** Investigation, Writing (review & editing). **Yun Chon:** Conceptualization, Investigation, Supervision, Writing (review & editing). **Julia Lynch:** Conceptualization, Funding acquisition, Investigation, Writing (review & editing).

## Declaration of Competing Interest

The authors declare that they have no known competing financial interests or personal relationships that could have appeared to influence the work reported in this paper.

## Appendix A Supplementary data

Supplementary Data A Statistical Analysis Plan Template.

Supplementary Data B Statistical Analysis Report Template.

Supplementary Data C Statistical Analysis Program Source Codes and Outputs.

Supplementary Data D Sample data (CSV format).
