# Supplementary material for: Statistical considerations on real time and extended controlled temperature conditions (ECTC) stability data analysis of vaccines
Source: Vaccine. 2023 Oct 6;41(42):6206–14. doi: 10.1016/j.vaccine.2023.08.012 (PMC10560890; doi:10.1016/j.vaccine.2023.08.012)
Supplement: Supplementary Data A — Statistical Analysis Plan Template. [file mmc1.docx]

STATISTICAL ANALYSIS PLAN

| Project Title: | Statistical Support to Acquire WHO CTC Label for [product name] |
| --- | --- |
| Study ID: | [XXXXXXX] |
|  |  |
|  |  |

**Prepared by:**

| **Name** | **Signature** | **Date** |
| --- | --- | --- |
| [Name of Statistician, MSc]  Project Statistician |  |  |

**Reviewed and Approved by:**

| **Name** | **Signature** | **Date** |
| --- | --- | --- |
| [Name of Head, Statistics, Ph.D.]  Head of Biostatistics |  |  |
| [Name of Project Lead, Ph.D.]  Project Lead |  |  |
| [Name of PI, MD]  Principal Investigator |  |  |

Revision History

| **Revision Date** | **Version** | **Reason** |
| --- | --- | --- |
| DDMMMYYYY | 1.0 | Initial version |

STATISTICAL ANALYSIS PLAN

For the project to Acquire WHO CTC Label for [product name]

**[MMM YYYYY]**

**Prepared by**

**[Statistics Department Name who**

**performed the statistical analysis]**

Revision History

| **Revision Date** | **Version** | **Reason** |
| --- | --- | --- |
| DDMMMYYYY | 1.0 | Initial version |
|  |  |  |

Abbreviations

| Abbreviation | Definition |
| --- | --- |
| ANCOVA | Analysis of Covariance |
| ºC | Degree Celsius |
| CTC | Controlled Temperature Chain |
| ECTC | Extended Controlled Temperature Conditions |
| GLM | General Linear Model |
| LL | lower limit |
| LOESS | Locally Weighted Smoothing |
| MRP | Minimum Release Potency |
| NA | Not Applicable |
| Q | Quarter |
| RT | Real Time |
| SAP | Statistical Analysis Plan |
| SAS | Statistical Analysis System |
| SD | Standard Deviation |
| t | Time |
| U | Uncertainty |
| WHO | World Health Organization |
| Y | Year |

**Table of Contents**

[1 INTRODUCTION 3](#_Toc80025891)

[2 OBJECTIVES 3](#_Toc80025892)

[2.1 Evaluation of vaccines for use under RT in normal cold chain 3](#_Toc80025893)

[2.2 Stability evaluation of vaccines for use under ECTC 3](#_Toc80025894)

[2.3 Calculation of Minimum Release Potency (MRP) using a statistical method 3](#_Toc80025895)

[3 STUDY OVERVIEW 4](#_Toc80025896)

[3.1 Study design 4](#_Toc80025897)

[3.2 Number of Lots 4](#_Toc80025898)

[3.3 Number of time points to follow-up 4](#_Toc80025899)

[4 STUDY ENDPOINTS 4](#_Toc80025900)

[4.1 Stability indicating parameters 4](#_Toc80025901)

[5 DEFINITIONS 4](#_Toc80025902)

[6 ANALYSIS SUBSET 6](#_Toc80025903)

[7 DATA SCREENING AND ACCEPTANCE 6](#_Toc80025904)

[7.1 Data Handling and Electronic Transfer of Data 6](#_Toc80025905)

[7.2 Handling of Missing and Incomplete Data 6](#_Toc80025906)

[7.3 Distributional Characteristics 6](#_Toc80025907)

[8 STATISTICAL ANALYSIS METHOD 6](#_Toc80025908)

[8.1 General Principles 7](#_Toc80025909)

[8.2 Data Exploration Graphically 7](#_Toc80025910)

[8.3 Linear Regression Diagnosis 7](#_Toc80025911)

[8.4 Test for Poolability 8](#_Toc80025912)

[8.5 Real condition storage data analysis 9](#_Toc80025913)

[8.6 ECTC exposure data analysis 9](#_Toc80025914)

[8.7 ECTC Exposure time and MRP calculation 9](#_Toc80025915)

[9 CHANGES FROM DATA COLLECTION PLAN 10](#_Toc80025916)

[10 LIST OF PLANNED TABLES, FIGURES AND LISTINGS [TFLs] 10](#_Toc80025917)

# INTRODUCTION

The purpose of this Statistical Analysis Plan (SAP) is to provide details of the statistical methodology and analyses to be performed as outlined in the guidelines titled “Guideline on the stability evaluation of vaccines for use under extended controlled temperature conditions” published by WHO (2015) and [translated in Korean by MFDS (2016) or proper national guidelines as necessary]. The scope of this plan includes the analysis that is planned and will be executed by the [Department of Statistics or proper department name].

# OBJECTIVES

The objectives are to evaluate two stability study data under real-time/real-condition and accelerated conditions and to calculate MRP for supporting the CTC label claim of [product name].

## Evaluation of vaccines for use under RT in normal cold chain

- To evaluate long-term storage stability of [product name], under real-time, real-condition in the normal cold chain (2 to 8ºC) with the available number of lots and follow-up period (the long-term follow-up period and number of lots are referred to the stability data collection plan prepared by [manufacturer name])
- To verify if the established shelf-life of [product name] is appropriate using a statistical method

## Stability evaluation of vaccines for use under ECTC

- To evaluate stability at the extended controlled temperature condition (40ºC) in number of lots of [product name] with different ages of storage
- To estimate how much exposure to ECTC condition could make a critical impact on the product stability

## Calculation of Minimum Release Potency (MRP) using a statistical method

- To calculate MRPs by statistical modeling using data from real-time/real-condition stability study and accelerated stability study

# STUDY OVERVIEW

According to the stability data collection plan, [antigen contents or proper stability-indicating parameter] are obtained from various lots at each time point.

## Study design

Refer to the data collection plan (See Section 10.1).

## Number of Lots

As per the data collection plan, a total of twenty-two lots are planned for stability studies.

- [24 lots or proper number of lots] for real time, real-condition: [6 lots per test set in each Q2Y1, Q4Y1, Q2Y2, and Q4Y2]
- [12 lots or proper number of lots] for ECTC condition: [3 lots per test set]

## Number of time points to follow-up

A maximum [24 months or proper months according to sheld-life] followed up for real-condition in normal cold chain and a maximum [14 or target] days follow-up for accelerated condition.

- [Seven or proper] time points for long-term stability: [0, 3, 6, 9, 12, 18, and 24] months
- [Six or proper] time points for accelerated condition stability: [0, 3, 7, 10, 12, and 14] days

# STUDY ENDPOINTS

## Stability indicating parameters

[Antigen content or proper stability indicating parameter]

# DEFINITIONS

The definitions given below apply to the terms used in the stability studies.

- Cold chain: A series of storage and transport links used for keeping and distributing vaccines in good condition until use according to the approved long term storage condition and shelf life. The typical temperature for the long term storage condition is 2 to 8ºC although other approved temperatures can be specified.
- WHO Controlled Temperature Chain (CTC) Programme: A specific approach to vaccine management that allows vaccines to be kept at temperatures above the long term storage condition (typically 2 to 8ºC) for a limited period of time under monitored and controlled conditions, as appropriate to the stability of the antigen. Current WHO programme conditions for CTC include a single exposure just prior to administration, tolerating ambient temperatures of at least 40ºC for a limited duration of at least three days, with these temperature and time conditions included in the approved label.
- Extended controlled temperature conditions (ECTC): Approved short-term temperature conditions above those defined for long-term storage, transportation and use, for a given product immediately prior to administration. Any temperatures above the approved long term storage in the cold chain could be considered for ECTC application. The development of the CTC terminology and the proposal of the alternative ECTC terminology is described in the Ottawa meeting report (2012).
- Real-time and real-condition stability studies: Studies on the physical, chemical, biological, biopharmaceutical and microbiological characteristics of a vaccine, during and up to the expected shelf-life and storage periods of samples under expected handling and storage conditions. The results are used to recommend storage conditions and to establish the shelf-life and/or the release specifications.
- Accelerated stability studies: Studies designed to determine the impact over time of exposure to temperatures higher than those recommended for storage on the physical, chemical, biological, biopharmaceutical and microbiological characteristics of a vaccine. When the accelerated temperature conditions are equivalent to or higher than the ECTC condition under evaluation, the accelerated stability data can be considered in support of the target ECTC conditions.
- Shelf-life: The period of time during which a vaccine, when stored under approved conditions, is expected to comply with the specifications. The shelf-life is determined by stability studies on a number of batches of the product and is used to establish the expiry date of each batch of a final product.
- Product release model: A model that describes the relationship between release and expiry specifications to ensure that the product will meet defined specifications throughout its shelf-life.
- Stability-indicating parameters: Quality parameters (direct or indirect indicators of vaccine efficacy or safety) that are sensitive to storage conditions. These parameters are used in stability studies to assure product quality throughout the shelf-life. Determination of these parameters should result in quantitative values with a detectable rate of change. Qualitative parameters such as sterility may also be considered but cannot be included in the statistical analysis.
- Stability of vaccine: The ability of a vaccine to retain its physical, chemical, biological and microbiological properties within specified limits to assure clinical performance throughout its shelf-life.
- Quality attributes: Physical, chemical, biological and microbiological attributes that can be defined, measured and continually monitored to ensure that final product outputs remain within acceptable quality limits.

# ANALYSIS SUBSET

The analysis set will include all collected data as planned and will be used for stability assessment under real-condition and ECTC, and MRP calculation.

# DATA SCREENING AND ACCEPTANCE

## Data Handling and Electronic Transfer of Data

The study data will be validated and entered electrically by [manufacturer name] and transferred to [Department of Biostatistics or proper department who performed the statistical analysis].

## Handling of Missing and Incomplete Data

The missing data due to planned missing time points will not be imputed.

## Distributional Characteristics

[Antigen content or proper stability indicating parameter] will be logarithmically transformed prior to statistical modeling in order to better approximate normality.

# STATISTICAL ANALYSIS METHOD

In principle, the linear regression model is considered according to the guidelines. However, other approaches are also considered if data are violated for linear regression model assumptions.

## General Principles

The normality of outcome measurements and linearity between outcomes and time points will be checked qualitatively and quantitatively. Since [six lots manufactured in each test set by 6-month interval for 2 years] are used for evaluation, the poolability of data is tested using either analysis of covariance (ANCOVA), general linear model (GLM), or mixed effect model, considering time and/or lot as covariates. The test of the differences in slopes and intercepts of regression lines among lots is conducted using a significance level of 0.25 to compensate for the expected low power of design due to the relatively limited sample size in a stability study. There are some rules for poolability of data. If the test rejects the hypothesis of equality of slopes, it is not considered appropriate to combine the data from all lots. The shelf-lives for individual batches in the stability can be estimated. The shortest estimate among the batches should be chosen as the shelf-life for all batches. If the tests for equality of slopes and equality of intercepts do not result in rejection at a level of significance of 0.25, the data from all batches can be combined. A single shelf-life can be estimated from the combined data. Each of ECTC data exposed at several different time points will be considered in the statistical model.

## Data Exploration Graphically

The [antigen contents] (outcome measurements) will be graphically explored and investigated if there exist similar intercepts ([antigen contents] at 0 months) and the same trend of slopes over time by a lot and the change patterns look non-linear over time due to suspicious values.

## Linear Regression Diagnosis

Log-transformed [antigen contents] is used for checking the following linear regression assumptions: linear relationship, influence, normality of residuals, homogeneity of variance of the residuals, and independence.

**Tests on nonlinearity:** The relationships between the predictor (time in months) and the [antigen contents] should be linear. Through the scatter plot between the outcome measurements and predictor (time) to see if nonlinearity is present. Added both lines of linear line and LOESS (Locally Weighted Smoothing) line to see the relationship between variables and foresee trends.

**Unusual and influence data:** There are three ways that an observation can be unusual: outlier (is an observation with large residual), leverage (an observation with extreme value on a predictor variable which affects the estimate of regression coefficient), and influence (influential if removing the observation substantially changes the estimate of coefficients, this can be thought of as the product of leverage and outlier-ness). The following table summarizes the general rules of thumb we use for these measures to identify unusual observations (where k is the number of predictors and n is the number of observations).

| Measure | Value |
| --- | --- |
| Leverage | >(2k+2)/n |
| Abs(studentized residual) | >2 |
| Cooks’D | >4/n |
| Abs(DFITS) | >2*sqrt(k/n) |
| Abs(DFBETA) | >2/sqrt(n) |

**Tests for normality of residuals:** In the Shapiro-Wilk W test for normality, the p-value is based on the assumption that the distribution is normal.

**Tests for heteroscedasticity:** One of the main assumptions for the ordinary least squares regression is the homogeneity of variance of the residuals. If the variance of the residuals is non-constant, then the residual variance is said to be heteroscedastic. There are graphical methods (plot the residuals versus fitted (predicted) values) and non-graphical methods (White test).

**Independence:** Since the data were collected on the same lots over time, the assumption of independence can be broken. In this situation, the errors for observations between adjacent time points will be more highly correlated than for observations more separated in time. Durbin-Watson’s d tests the null hypothesis that the residuals are not linearly auto-correlated. As a rule of thumb values of 1.5 < d < 2.5 show that it is not auto-correlation.

## Test for Poolability

Prior to poolability test, it will be checked the normality of outcome measurements. Upon normality test and reducing the effect of outliers in the linear regression model, all outcome measurements will be natural logarithmically transformed for analysis.

According to the following criteria (Ref. ICH Q1E. Evaluation for Stability Data. B3.2. Tests for Poolability), the test of equality of slope and test of intercept will determine if the combined data will be used in the model.

| Equality of Slope p-value | Equality of Intercept p-value | Final Reduced Model | | |
| --- | --- | --- | --- | --- |
|  |  | Slope | Intercept | Description |
| ≤ 0.25 | NA | Separate | Separate | Not Parallel |
| > 0.25 | ≤ 0.25 | Common | Separate | Parallel |
| > 0.25 | > 0.25 | Common | Common | One Line |

## Real condition storage data analysis

Each [antigen contents] over time will be explored and investigated graphically. As following linear regression diagnosis and poolability test, linear regression model will be determined appropriately and assess the stability. Each mean regression line and its lower bound of 95% confidence bound for mean and individual predicted value will be used for assessment.

## ECTC exposure data analysis

Each [antigen contents] over time will be explored and investigated graphically. As following linear regression diagnosis and poolability test, linear regression model will be determined appropriately and assess the stability.

## ECTC Exposure time and MRP calculation

To estimate the loss of [antigen contents] under both long term storage under 2-8ºC condition and a short period under 40ºC conditions with 95% confidence, we will use the linear regression model and the formula described in Section 5 of WHO Guideline on the stability evaluation of vaccines for use under extended controlled temperature conditions. Using a worst-case lot at release (calculated MRP), estimate appropriate exposure time (days) at ECTC temperature.

| **Total decay of [antigen contents] at 2-8ºC over 24 months, plus 3 (or 7, 10, 12, and 14) days at 40ºC:** |
| --- |
| = [antigen contents] at release (worst-case lot) – [antigen contents] at the end of storage (2-8ºC plus 40ºC)  = [antigen contents] at release (worst-case lot) –  exp[log(worst-case lot at release) + (t_2_8_ ∙ b_2_8_ + t_ECTC_ ∙ b_ECTC_ – U)],  where  t_2_8_ is time at 2-8ºC,  b_2_8_ is decay slope (a negative, or zero if positive) at temperature 2-8ºC,  t_ECTC_ is time at 40ºC,  b_ECTC_ decay slope (a negative, or zero if positive) at temperature 40ºC,  U is combined uncertainty at 2-8 ºC and uncertainty followed by exposure days at 40ºC |

| **MRP through expiry including ECTC exposure:** |
| --- |
| = LL – [antigen contents] at the end of storage (2-8ºC plus 40ºC),  => exp[log(LL) – (t_2_8_ ∙ b_2_8_ + t_ECTC_ ∙ b_ECTC_ – U)] ,  where  LL: minimum potency  t_2_8_ is time at 2-8ºC,  b_2_8_ is decay slope (a negative, or zero if positive) at temperature 2-8ºC,  t_ECTC_ is time at 40ºC,  b_ECTC_ decay slope (a negative, or zero if positive) at temperature 40ºC,  U is combined uncertainty at 2-8ºC and uncertainty followed by exposure days at 40ºC |

# CHANGES FROM DATA COLLECTION PLAN

According to the data collection plan (DDMMMYYYY), the number of lots and data collection time points were revised in the SAP.

# LIST OF PLANNED TABLES, FIGURES AND LISTINGS [TFLs]

The planned tables, figures, and listings will be provided.

10. STATISTICAL ANALYSIS RESULT

10.1 Stability Data Collection

Table 10.1.1. Stability data collection scheme

Table 10.1.2. Distribution of RT stability data time points by lot number

Table 10.1.3. Distribution of ECTC stability data time points by lot number

Table 10.1.4. Descriptive statistics of RT stability data by time point

Table 10.1.5. Descriptive statistics of ECTC stability data by data time point

Figure 10.1.1. Mean and SD of [antigen contents] under RT over time

Figure 10.1.2. Mean and SD of [antigen contents] under ECTC over time

10.2 Evaluation of vaccines for use RT in normal cold chain

Figure 10.2.1. [Antigen contents] trend under RT over time

10.3 Stability evaluation of vaccines for use under ECTC

Figure 10.3.1. [Antigen contents] under ECTC over time

10.4 ECTC Exposure time and MRP calculation

Table 10.4.1. Statistical analysis of [antigen contents] at 2-8°C

Table 10.4.2. Statistical analysis of [antigen contents] at 40°C

Table 10.4.3. MRP Calculation: Pooled ECTC data and by exposed month
