# Supplementary material for: Statistical considerations on real time and extended controlled temperature conditions (ECTC) stability data analysis of vaccines
Source: Vaccine. 2023 Oct 6;41(42):6206–14. doi: 10.1016/j.vaccine.2023.08.012 (PMC10560890; doi:10.1016/j.vaccine.2023.08.012)
Supplement: Supplementary Data B — Statistical Analysis Report Template. [file mmc2.docx]

STATISTICAL ANALYSIS REPORT

For the project to Acquire WHO CTC Label for [product name]

**[MMM YYYYY]**

**Prepared by**

**[Statistics Department Name who**

**performed the statistical analysis]**

Revision History

| **Revision Date** | **Version** | **Reason** |
| --- | --- | --- |
| DDMMMYYYY | 1.0 | Initial version |
|  |  |  |

Abbreviations

| Abbreviation | Definition |
| --- | --- |
| ANCOVA | Analysis of Covariance |
| ºC | Degree Celsius |
| CTC | Controlled Temperature Chain |
| D | Day |
| ECTC | Extended Controlled Temperature Conditions |
| GLM | General Linear Model |
| LEU | Lipopolysaccharide ELISA Units |
| LL | lower limit |
| LOESS | Locally Weighted Smoothing |
| M | Month |
| MRP | Minimum Release Potency |
| NA | Not Applicable |
| Q | Quarter |
| RT | Real Time |
| SAP | Statistical Analysis Plan |
| SAS | Statistical Analysis System |
| SD | Standard Deviation |
| t | Time |
| U | Uncertainty |
| WHO | World Health Organization |
| Y | Year |

**Table of Contents**

[10 Statistical Analysis Result 5](#_Toc80022360)

[10.1 Stability Data collection 6](#_Toc80022361)

[10.2 Evaluation of vaccines for use under RT in normal cold chain 10](#_Toc80022362)

[10.3 Stability evaluation of vaccines for use under ECTC 11](#_Toc80022363)

[10.4 ECTC Exposure time and MRP calculation 12](#_Toc80022364)

**List of Tables**

[Table 10.1.1. Stability data collection scheme 6](#_Toc80022365)

[Table 10.1.2. Distribution of RT stability data time points by lot number 6](#_Toc80022366)

[Table 10.1.3. Distribution of ECTC stability data time points by lot number 7](#_Toc80022367)

[Table 10.1.4. Descriptive statistics of RT stability data by time point 8](#_Toc80022368)

[Table 10.1.5. Descriptive statistics of ECTC stability data by data time point 9](#_Toc80022369)

[Table 10.4.1. Statistical analysis of [antigen contents] under RT 12](#_Toc80022370)

[Table 10.4.2. Statistical analysis of [antigen contents] under ECTC 13](#_Toc80022371)

[Table 10.4.3. MRP Calculation: Pooled ECTC data and by exposed month 14](#_Toc80022372)

**List of Figures**

[Figure 10.1.1. Mean and SD of [antigen contents] under RT over time 9](#_Toc80022373)

[Figure 10.1.2. Mean and SD of [antigen contents] under ECTC over time. 9](#_Toc80022374)

[Figure 10.2.1. [Antigen contents] trend under RT over time 11](#_Toc80022375)

[Figure 10.3.1. [Antigen contents] under ECTC over time 12](#_Toc80022376)

# Statistical Analysis Result

The statistical analysis of the long-term normal storage stability evaluation under normal condition and short-term storage stability under extended controlled temperature conditions of [product name] was performed according to the planned statistical analysis (SAP version 1.0 dated DDMMMYYYY). In addition, the minimum release potency (MRP) was calculated using the given shelf-life and the rate of decay of the potency over both the long-term storage temperature and the extended controlled temperature conditions to ensure that the [product name] is above the lower limit of potency.

The stability data collection plan (dated DDMMMYYYY) was developed, and stability data was collected as planned.

The [antigen contents or proper parameter which was considered for stability assessment] as stability indicating parameter was descriptively summarized and graphically explored by time points and [antigen name] and used for estimation of mean potency and lower limit of potency over time in each [antigen name]. The linear regression model by checking normality and linearity assumptions was applied to evaluate stability of potency over time. The [antigen or proper parameter] content was logarithmically transformed prior to statistical modeling in order to better approximate normality. The Poolability test result using general linear model (GLM) and criteria (ICH Q1E. Evaluation for Stability Data. B3.2. Tests for Poolability) was suggested separate intercept and separate slope. Therefore, individual mean regression line and 95% confidence interval by lot was calculated from the linear regression model. The statistical analysis was performed using [SAS version 9.4 (SAS Institute, Cary, NC, USA) or The R Project for Statistical Computing].

The estimated mean regression line and lower limit of 95% confidence interval of [antigen or proper parameter] content was above approved lower limit (LL) under both the long-term storage temperature and the extended controlled temperature conditions. The calculated minimum release potency given [24 or 36 or proper shelf-life] months shelf-life and [3 or 14 or target days] days at the extended controlled temperature conditions was [XXX LEU or proper unit] for each [antigens or proper parameters], respectively.

## Stability Data collection

The planned stability data collection scheme was presented in Table 10.1.1. The stability data time points of real time/real condition and extended controlled temperature condition were presented in Table 10.1.2 and 10.1.3. The descriptive statistics of real-time and real-condition stability and ECTC data by serotype and data time point was presented in Table 10.1.4 and Table 10.1.5.

Table 10.1.1. Stability data collection scheme [revise it as appropriate]

| Test set | Stability testing method | Number of Batches | Manufacture Date | Testing timepoints | Test Completion Date |
| --- | --- | --- | --- | --- | --- |
| A | RT | 3 Batches | Q2Y1 | Month 0, 3, 6, 9, 12, 18, 24 | Q2Y1 |
|  | ECTC | 3 Batches | Q2Y1 | Day 0, 3, 7, 10, 12, 14  at each exposed Month 0, 6, 12, 24 | Q2Y1 |
| B | RT | 3 Batches | Q4Y1 | Month 0, 3, 6, 9, 12, 18, 24 | Q4Y1 |
|  | ECTC | 3 Batches | Q4Y1 | Day 0, 3, 7, 10, 12, 14  at each exposed Month 0, 6, 12 | Q4Y1 |
| C | RT | 3 Batches | Q2Y2 | Month 0, 3, 6, 9, 12 18, 24 | Q2Y2 |
|  | ECTC | 3 Batches | Q2Y2 | Day 0, 3, 7, 10, 12, 14  at each exposed Month 0, 6, 12 | Q2Y2 |
| D | RT | 3 Batches | Q4Y2 | Month 0, 3, 6, 9, 12 18, 24 | Q4Y2 |
|  | ECTC | 3 Batches | Q4Y2 | Day 0, 3, 7, 10, 12, 14  at each exposed Month 0, 6, 12 | Q4Y2 |

[Note] RT: Real time and Real conditions in the normal cold chain (2 to 8ºC); ECTC: Accelerated storage conditions (40ºC); Q: Quarter; Y: Year.

Table 10.1.2. Distribution of RT stability data time points by lot number [revise it as appropriate]

| **Batches/Lots number** | **Data time points (Months) for real-time stability** | | | | | | | |
| --- | --- | --- | --- | --- | --- | --- | --- | --- |
|  | **M0** | **M3** | **M6** | **M9** | **M12** | **M18** | **M24** | **Total** |
| Batch01 | 1 | 1 | 1 | 1 | 1 | 1 | 1 | 7 |
| Batch02 | 1 | 1 | 1 | 1 | 1 | 1 | 1 | 7 |
| Batch03 | 1 | 1 | 1 | 1 | 1 | 1 | 1 | 7 |
| Batch04* | 1 | 0 | 1 | 0 | 1 | 0 | 1 | 4 |
| Batch05* | 1 | 0 | 1 | 0 | 1 | 0 | 1 | 4 |
| Batch06* | 1 | 0 | 1 | 0 | 1 | 0 | 1 | 4 |
| Batch07 | 1 | 1 | 1 | 1 | 1 | 1 | 1 | 7 |
| Batch08 | 1 | 1 | 1 | 1 | 1 | 1 | 1 | 7 |
| Batch09 | 1 | 1 | 1 | 1 | 1 | 1 | 1 | 7 |
| Batch10* | 1 | 0 | 1 | 0 | 1 | 0 | 0 | 3 |
| Batch11* | 1 | 0 | 1 | 0 | 1 | 0 | 0 | 3 |
| Batch12* | 1 | 0 | 1 | 0 | 1 | 0 | 0 | 3 |
| Batch13 | 1 | 1 | 1 | 1 | 1 | 1 | 1 | 7 |
| Batch14 | 1 | 1 | 1 | 1 | 1 | 1 | 1 | 7 |
| Batch15 | 1 | 1 | 1 | 1 | 1 | 1 | 1 | 7 |
| Batch16* | 1 | 0 | 1 | 0 | 1 | 0 | 0 | 3 |
| Batch17* | 1 | 0 | 1 | 0 | 1 | 0 | 0 | 3 |
| Batch18* | 1 | 0 | 1 | 0 | 1 | 0 | 0 | 3 |
| Batch19 | 1 | 1 | 1 | 1 | 1 | 1 | 1 | 7 |
| Batch20 | 1 | 1 | 1 | 1 | 1 | 1 | 1 | 7 |
| Batch21 | 1 | 1 | 1 | 1 | 1 | 1 | 1 | 7 |
| Batch22* | 1 | 0 | 1 | 0 | 1 | 0 | 0 | 3 |
| Batch23* | 1 | 0 | 1 | 0 | 1 | 0 | 0 | 3 |
| Batch24* | 1 | 0 | 1 | 0 | 1 | 0 | 0 | 3 |
| **Total** | **24** | **12** | **24** | **12** | **24** | **12** | **15** | **123** |

[Note] *The lots used in ECTC were included for RT.

Table 10.1.2 presented actual status of the long-term stability data under real-time and real-condition in normal cold chain (2 to 8ºC) collected at different time points (months) for 24 months storage from 24 lots manufactured in Q2Y1, Q4Y1, Q2Y2, and Q4Y2. The 12 more batch/lot numbers used in ECTC stability testing were included in RT stability data analysis in order to increase the number of lots and number of data points for statistical modelling.

Table 10.1.3. Distribution of ECTC stability data time points by lot number [revise it as appropriate]

| **Batches** | **Exposed Month** | **Testing timepoints (days)** | | | | | | |
| --- | --- | --- | --- | --- | --- | --- | --- | --- |
|  |  | **D0** | **D3** | **D7** | **D10** | **D12** | **D14** | **Total** |
| Batch04 | M0 | 1 | 1 | 1 | 1 | 1 | 1 | 6 |
| Batch05 | M0 | 1 | 1 | 1 | 1 | 1 | 1 | 6 |
| Batch06 | M0 | 1 | 1 | 1 | 1 | 1 | 1 | 6 |
| Batch10 | M0 | 1 | 1 | 1 | 1 | 1 | 1 | 6 |
| Batch11 | M0 | 1 | 1 | 1 | 1 | 1 | 1 | 6 |
| Batch12 | M0 | 1 | 1 | 1 | 1 | 1 | 1 | 6 |
| Batch16 | M0 | 1 | 1 | 1 | 1 | 1 | 1 | 6 |
| Batch17 | M0 | 1 | 1 | 1 | 1 | 1 | 1 | 6 |
| Batch18 | M0 | 1 | 1 | 1 | 1 | 1 | 1 | 6 |
| Batch22 | M0 | 1 | 1 | 1 | 1 | 1 | 1 | 6 |
| Batch23 | M0 | 1 | 1 | 1 | 1 | 1 | 1 | 6 |
| Batch24 | M0 | 1 | 1 | 1 | 1 | 1 | 1 | 6 |
| **Sub-total** | **M0** | **12** | **12** | **12** | **12** | **12** | **12** | **72** |
| Batch04 | M6 | 1 | 1 | 1 | 1 | 1 | 1 | 6 |
| Batch05 | M6 | 1 | 1 | 1 | 1 | 1 | 1 | 6 |
| Batch06 | M6 | 1 | 1 | 1 | 1 | 1 | 1 | 6 |
| Batch10 | M6 | 1 | 1 | 1 | 1 | 1 | 1 | 6 |
| Batch11 | M6 | 1 | 1 | 1 | 1 | 1 | 1 | 6 |
| Batch12 | M6 | 1 | 1 | 1 | 1 | 1 | 1 | 6 |
| Batch16 | M6 | 1 | 1 | 1 | 1 | 1 | 1 | 6 |
| Batch17 | M6 | 1 | 1 | 1 | 1 | 1 | 1 | 6 |
| Batch18 | M6 | 1 | 1 | 1 | 1 | 1 | 1 | 6 |
| Batch22 | M6 | 1 | 1 | 1 | 1 | 1 | 1 | 6 |
| Batch23 | M6 | 1 | 1 | 1 | 1 | 1 | 1 | 6 |
| Batch24 | M6 | 1 | 1 | 1 | 1 | 1 | 1 | 6 |
| **Sub-total** | **M6** | **12** | **12** | **12** | **12** | **12** | **12** | **72** |
| Batch04 | M12 | 1 | 1 | 1 | 1 | 1 | 1 | 6 |
| Batch05 | M12 | 1 | 1 | 1 | 1 | 1 | 1 | 6 |
| Batch06 | M12 | 1 | 1 | 1 | 1 | 1 | 1 | 6 |
| Batch10 | M12 | 1 | 1 | 1 | 1 | 1 | 1 | 6 |
| Batch11 | M12 | 1 | 1 | 1 | 1 | 1 | 1 | 6 |
| Batch12 | M12 | 1 | 1 | 1 | 1 | 1 | 1 | 6 |
| Batch16 | M12 | 1 | 1 | 1 | 1 | 1 | 1 | 6 |
| Batch17 | M12 | 1 | 1 | 1 | 1 | 1 | 1 | 6 |
| Batch18 | M12 | 1 | 1 | 1 | 1 | 1 | 1 | 6 |
| Batch22 | M12 | 1 | 1 | 1 | 1 | 1 | 1 | 6 |
| Batch23 | M12 | 1 | 1 | 1 | 1 | 1 | 1 | 6 |
| Batch24 | M12 | 1 | 1 | 1 | 1 | 1 | 1 | 6 |
| **Sub-total** | **M12** | **12** | **12** | **12** | **12** | **12** | **12** | **72** |
| Batch04 | M24 | 1 | 1 | 1 | 1 | 1 | 1 | 6 |
| Batch05 | M24 | 1 | 1 | 1 | 1 | 1 | 1 | 6 |
| Batch06 | M24 | 1 | 1 | 1 | 1 | 1 | 1 | 6 |
| **Sub-total** | **M24** | **3** | **3** | **3** | **3** | **3** | **3** | **18** |

Table 10.1.3 presented actual status of stability data under extended controlled temperature condition (40 ºC) collected at different time points (days) for 14 days from 12 lots manufactured in Q2Y1, Q4Y1, Q2Y2, and Q4Y2. There were 4 different exposed months: very early storage period at 0 and 6 months, medium storage period at 12 months, and very late storage period at 24 months. [Depending on the lot variation at 24 months, an increased number of batches need to be considered.]

Table 10.1.4. Descriptive statistics of RT stability data by time point [revise it as appropriate]

| **Observed data time point (Months)** | **Number of observed data** | **Antigen content (Mean ± SD)** |
| --- | --- | --- |
| M0 | 24 | 924.12 ± 23.55 |
| M3 | 12 | 908.88 ± 23.5 |
| M6 | 24 | 894.6 ± 23.73 |
| M9 | 12 | 873.82 ± 27.73 |
| M12 | 24 | 869.13 ± 22.96 |
| M18 | 12 | 859.29 ± 19.16 |
| M24 | 15 | 845.01 ± 13.11 |

Table 10.1.4 descriptively presented the mean and standard deviation (SD) of observed [antigen contents] data under real time and real condition, which were above acceptable criteria (XXX LEU) at each time point for 24 months.

Figure 10.1.1. Mean and SD of [antigen contents] under RT over time


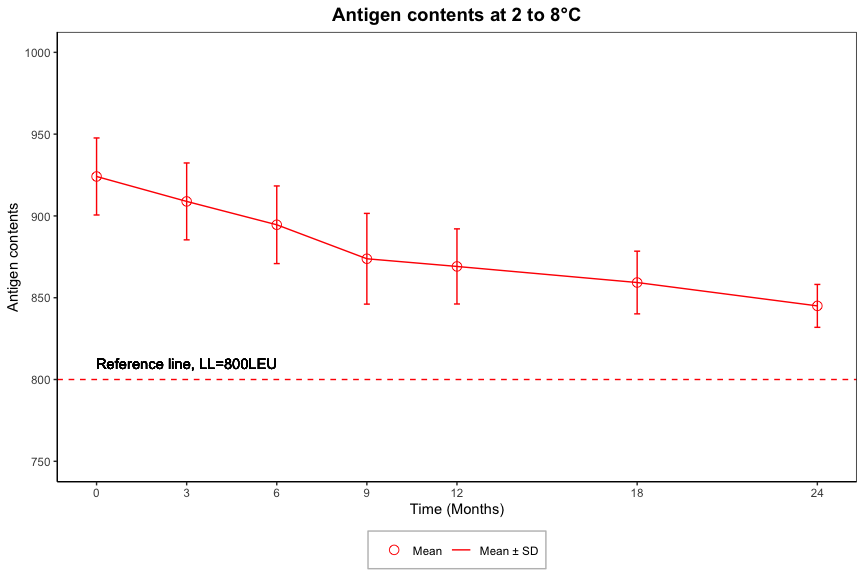


Figure 10.1.1 graphically presented the mean and standard deviation (SD) of observed [antigen contents] data under real time and real condition, which were above acceptable criteria (XXX LEU) at each time point for 24 months. The [antigen contents] data were stable for the first 12 months and then stepped down at 15 months and stable after 15 months.

Figure 10.1.2. Mean and SD of [antigen contents] data under ECTC over time.

Table 10.1.5. Descriptive statistics of ECTC stability data by data time point [revise it as appropriate]

| **Observed data time point (Months)** | **Number of observed data** | **Antigen content (Mean ± SD)** |
| --- | --- | --- |
| D0 | 39 | 904.04 ± 18.03 |
| D3 | 39 | 892.63 ± 22.01 |
| D7 | 39 | 876.02 ± 20.23 |
| D10 | 39 | 875.8 ± 20.85 |
| D12 | 39 | 865.12 ± 25.79 |
| D14 | 39 | 850.88 ± 23.37 |

Table 10.1.5 descriptively presented the mean and standard deviation (SD) of observed [antigen contents] data under ECTC, which were above acceptable criteria (XXX LEU) at each time point for 24 months.

Figure 10.1.2. Mean and SD of [antigen contents] under ECTC over time


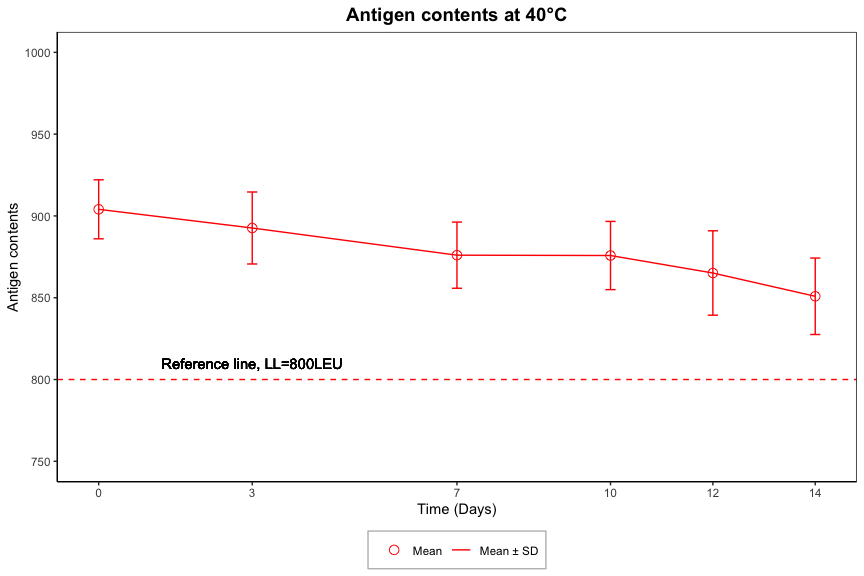


Figure 10.1.2 graphically presented the mean and SD of observed [antigen contents], which were stable and above acceptable criteria (XXX LEU) at each time point for 14 days.

## Evaluation of vaccines for use under RT in normal cold chain

Figure 10.2.1 presented the observed [antigen contents] and predicted mean regression linear lines with its 95% confidence intervals. It showed that all observed [antigen contents] and the lower bound of 95% confidence intervals were above acceptable criteria (XXX LEU) over the time period. It showed stable [antigen contents] over time within lots and among lots. The estimated mean regression lines showed a negative value of slope.

Figure 10.2.1. [Antigen contents] trend under RT over time


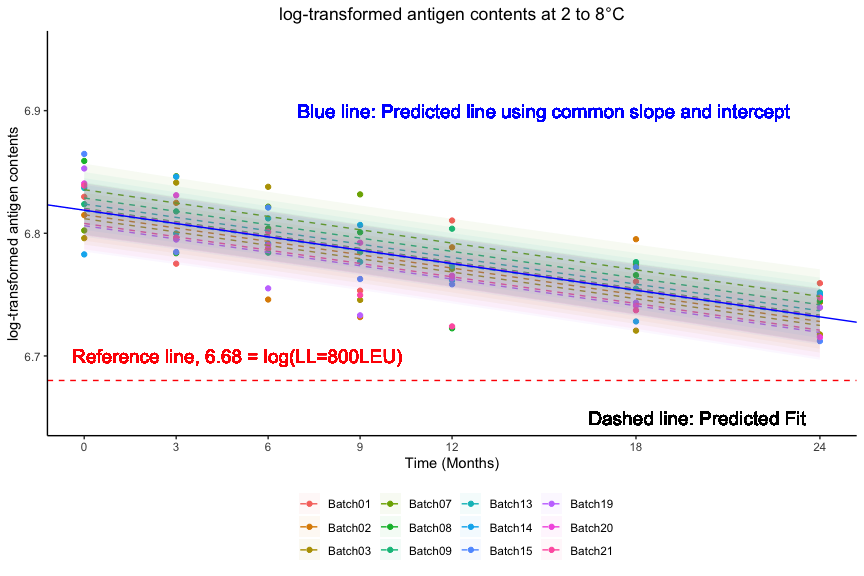


## Stability evaluation of vaccines for use under ECTC

Figure 10.3.1 presented the observed [antigen contents] and predicted mean regression linear lines with its 95% confidence intervals. It showed that all observed [antigen contents] and the lower bound of 95% confidence intervals were above acceptable criteria (XXX LEU) over the time period. It showed stable [antigen contents] over time within lots and among lots. The estimated mean regression lines showed a negative value of slope.

Figure 10.3.1. [Antigen contents] under ECTC over time


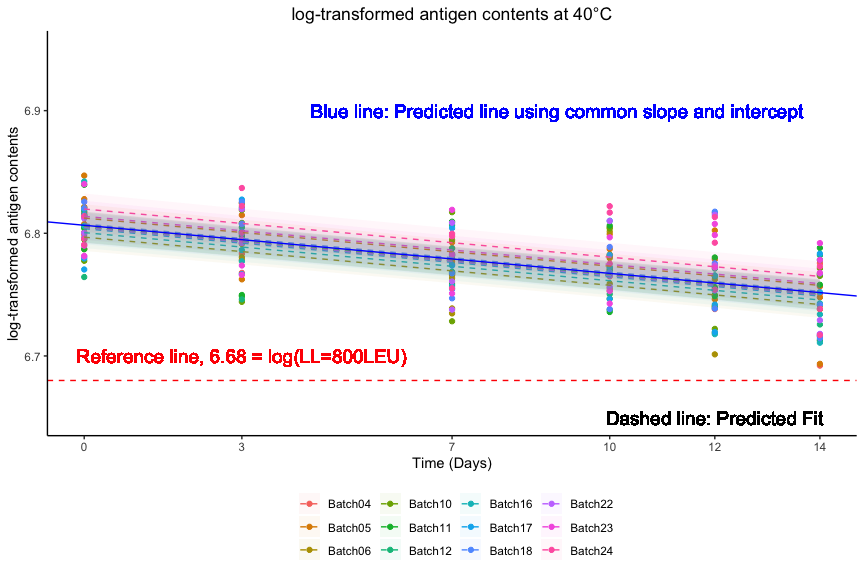


## ECTC Exposure time and MRP calculation

Using two linear regressions for each normal temperature condition and extended controlled temperature condition, the MRP was calculated for each serotype. The formula described in section 5 in WHO guideline on the stability evaluation of vaccines for use under ECTC was used. The following parameters were considered for the MRP calculation:

- Shelf-life at 2-8°C: [24 or 36 or appropriate shelf-life] months
- ECTC exposure days at 40°C: [3 or 14 or target] days
- Time period of normal condition storage before ECTC exposure: 0, 6, 12, and 24 months

Table 10.4.1. Statistical analysis of [antigen contents] under RT

| **Batch number** | **Data set**  **(unit: months)** | **Shelf-life (months)** | **Pooled slope**  **(per month)** | **SE of slope** | **Residual STD** | **Uncertainty** |
| --- | --- | --- | --- | --- | --- | --- |
| **All 24 lots** | 0-24M | 24 | -0.003723 | 0.000377 | 0.0271 | 0.0469 |

Table 10.4.2. Statistical analysis of [antigen contents] under ECTC

| **Data set** | **Days at 40°C** | **Pooled slope (per months)** | **SE of slope** | **Residual STD** | **Combined uncertainty*** | **MRP** |
| --- | --- | --- | --- | --- | --- | --- |
| Pooled 12 lots (D0-D14) | 3 | -0. 117480 | 0. 009942 | 0. 0250 | 0. 0456 | 924 |
| Pooled 12 lots (D0-D14) | 14 | -0. 117480 | 0. 009942 | 0. 0250 | 0. 0462 | 966 |
| Pooled 12 lots (D0-D14) exposed at M0 | 3 | -0. 118629 | 0. 018841 | 0. 0263 | 0. 0457 | 925 |
| Pooled 12 lots (D0-D14) exposed at M0 | 14 | -0. 118629 | 0. 018841 | 0. 0263 | 0. 0479 | 968 |
| Pooled 12 lots (D0-D14) exposed at M6 | 3 | -0. 126544 | 0. 018649 | 0.0260 | 0. 0457 | 925 |
| Pooled 12 lots (D0-D14) exposed at M6 | 14 | -0. 126544 | 0. 018649 | 0.0260 | 0. 0478 | 971 |
| Pooled 12 lots (D0-D14) exposed at M12 | 3 | -0. 102528 | 0. 017323 | 0. 0242 | 0.0470 | 923 |
| Pooled 12 lots (D0-D14) exposed at M12 | 14 | -0. 102528 | 0. 017323 | 0. 0242 | 0. 0488 | 960 |
| Pooled 3 lots (D0-D14) exposed at M24 | 3 | -0. 136440 | 0. 034428 | 0. 0240 | 0. 0460 | 926 |
| Pooled 3 lots (D0-D14) exposed at M24 | 14 | -0. 136440 | 0. 034428 | 0. 0240 | 0. 0527 | 981 |

[Note] * Uncertainty at RT for 24 months followed by 3 and 14 days at ECTC; M” Month; D: Day.

Using ECTC data from pooled all [24] lots, an [YYY LEU] shown below was derived as the MRP of [antigen contents] at 2-8°C for 24 months followed by 3 and 14 days at 40 °C. A similar trend of MRP for various ECTC exposure days: 3 and 14 days was derived using ECTC data exposed at each 0, 3, 6, 12, and 24.

**MRP** = LL – [antigen contents] at the end of storage (2-8 ºC plus 40 ºC),

[on natural log-transformed data]

= exp[log(LL**)** – (t_2_8_ ∙ b_2_8_ + t_ECTC_ ∙ b_ECTC_ – U)]

Using 24 months shelf-life and 3 Days exposed at 40ºC,

MRP = exp[log(800) – (24*(-0.003723¹) + (3/30)*( -0. 11748) – 0. 0456)]

= **924**

Using 24 months shelf-life and 14 Days exposed at 40ºC,

MRP = exp[log(800) – (24*(-0.003723¹) + (14/30)*( -0. 11748) – 0. 0462)]

= **966**

where,

t_2_8_ is time in months at 2-8°C, b_2_8_ is decay slope at temperature 2-8°C,

t_ECTC_ is time in months at 40°C, b_ECTC_ decay slope at temperature 40°C,

U is combined uncertainty at 2-8°C and uncertainty followed by exposure days at 40°C

The decay slopes are anticipated to have a negative value. However, the stability data under real-time and real-condition showed a positive decay slope. The positive decay slope (b_2_8_) from real-time and real-condition was replaced with zero (0) value according to the guideline T the decay slope (bi) must be negative value is decay slope (a negative number, or zero if positive) at temperature i (“WHO guideline on the stability evaluation of vaccines for use under ECTC,” 2015).

Finally, Table 10.4.3 summarized the calculated MRP by antigen on various ECTC exposure period and 24 months shelf-life. The MRP was very similar on various ECTC exposed time points and various exposure period from 3 days up to 14 days. The shorter shelf-life provided lower MRP than longer shelf-life.

Table 10.4.3. MRP Calculation: Pooled ECTC data and by exposed month

| **Antigen** | **LL**  **(LEU)** | **Number of lots for RT and ECTC** | **Data set**  **(2-8°C, 40°C)** | **Shelf-life**  **(months)** | **ECTC exposed at month** | **MRP (LEU), Days at 40ºC** | |
| --- | --- | --- | --- | --- | --- | --- | --- |
|  |  |  |  |  |  | **3 days** | **14 days** |
| Antigen | 800 | 24 and 12 | M0-M24, D0-D14 | 24 | Pooled | 924 | 966 |
| Antigen | 800 | 24 and 12 | M0-M24, D0-D14 | 24 | M0 | 925 | 968 |
| Antigen | 800 | 24 and 12 | M0-M24, D0-D14 | 24 | M6 | 925 | 971 |
| Antigen | 800 | 24 and 12 | M0-M24, D0-D14 | 24 | M12 | 923 | 960 |
| Antigen | 800 | 24 and 3 | M0-M24, D0-D14 | 24 | M24 | 926 | 981 |
