# Supplementary material for: Statistical considerations on real time and extended controlled temperature conditions (ECTC) stability data analysis of vaccines
Source: Vaccine. 2023 Oct 6;41(42):6206–14. doi: 10.1016/j.vaccine.2023.08.012 (PMC10560890; doi:10.1016/j.vaccine.2023.08.012)
Supplement: Supplementary Data C — Statistical Analysis Program Source Codes and Outputs. [file mmc3.docx]

Statistical Analysis Program Source Codes and Outputs

YYYY-MM-DD

# Introduction

This section provides sample data analysis including R program codes and its outputs as an example for the exercise.

# Prerequisites

We assume you have already installed R (<https://www.r-project.org>) and the RStudio (<https://www.rstudio.com>).

## Installing an R and R Package

Make sure you have installed the following R packages:

library(plyr)
library(tidyr)
library(summarytools)
library(kableExtra)
library(ggplot2)
library(car)
library(lmtest)
library(gvlma)
library(olsrr)
library(rstatix)
library(broom)

| Library name | Package link |
| --- | --- |
| **library**(plyr) | <https://cran.r-project.org/web/packages/plyr/plyr.pdf> |
| **library**(tidyr) | <https://cran.r-project.org/web/packages/tidyr/tidyr.pdf> |
| **library**(lmtest) | <https://cran.r-project.org/web/packages/lmtest/lmtest.pdf> |
| **library**(summarytools) | <https://cran.r-project.org/web/packages/summarytools/summarytools.pdf> |
| **library**(kableExtra) | <https://cran.r-project.org/web/packages/kableExtra/kableExtra.pdf> |
| **library**(ggplot2) | <https://cran.r-project.org/web/packages/ggplot2/ggplot2.pdf> |
| **library**(car) | <https://cran.r-project.org/web/packages/car/car.pdf> |
| **library**(gvlma) | <https://cran.r-project.org/web/packages/gvlma/gvlma.pdf> |
| **library**(olsrr) | <https://cran.r-project.org/web/packages/olsrr/olsrr.pdf> |
| **library**(rstatix) | <https://cran.r-project.org/web/packages/rstatix/rstatix.pdf> |
| **library**(broom) | <https://cran.r-project.org/web/packages/broom/broom> |

## Set the working directory

First, let’s make sure that you set your working directory. Rename “Your working directory name”.

# setwd("C:/Your working directory name")

Save as sample data (Sample_Data.csv) file under working directory. And create new folders name as **Table** and **Figure** under your working directory to save output files.

## Data preparation

### Data Import

We will use the built in function, read.csv(), which reads the data in as a data frame. And check number of records and look at the first six rows using nrow() and head().

org_data <- read.csv("Sample_Data.csv")
nrow(org_data)

## [1] 636

head(org_data)

## method batch nmonth ndays emonth repl antigen
## 1 RT Batch01 0 0 NA 1 928.43
## 2 RT Batch01 0 0 NA 2 921.51
## 3 RT Batch01 3 0 NA 1 883.60
## 4 RT Batch01 3 0 NA 2 868.14
## 5 RT Batch01 6 0 NA 1 913.88
## 6 RT Batch01 6 0 NA 2 859.39

tail(org_data)

## method batch nmonth ndays emonth repl antigen
## 631 ECTC Batch24 0.47 14 0 1 813.04
## 632 ECTC Batch24 0.47 14 0 2 875.33
## 633 ECTC Batch24 0.47 14 6 1 899.73
## 634 ECTC Batch24 0.47 14 6 2 848.06
## 635 ECTC Batch24 0.47 14 12 1 826.09
## 636 ECTC Batch24 0.47 14 12 2 826.65

| Var Name | Description |
| --- | --- |
| method | Stability testing method; RT: Real-time Real conditions in normal cold chain (2 to 8ºC) / ECTC: Accelerated storage conditions (40ºC) |
| batch | Batch number |
| nmonth | Month |
| ndays | Days |
| emonth | Exposed month |
| repl | Replicate number |
| antigen | Antigen contents (LEU/mL) |

### Calculate mean per group

The antigen means calculated by the ddply() per method, batch, nmonth (ndays), and exposed month for ECTC are used for analysis.

single_data <- ddply(org_data, .(method, batch, nmonth, ndays, emonth), summarize, antigen=mean(antigen))

### Selecting Observations based on variable values

Two data sets, RT and ECTC, by method will be used for analysis. Here, we can make new data set using subset().

**Real-time stability data set**

RT <- subset(single_data,method=='RT')

**ECTC stability data set**

ECTC <- subset(single_data,method=='ECTC')

# Stability Data collection

## Distribution of real-time stability data time points by batch

We will see the data distribution using table().

single_data$month <- ifelse(single_data$method=="RT",single_data$nmonth,single_data$emonth)
lbatch <- ddply(single_data, .(method, batch, month), summarize, antigen=mean(antigen))
table <- table(lbatch$batch,lbatch$month)
table_1 <- cbind(table,Total=margin.table(table, 1))
table_2 <- cbind(t(margin.table(table, 2)) ,Total=sum(margin.table(table, 1)))
Dist <- rbind(table_1,Total = table_2)
Dist

## 0 3 6 9 12 18 24 Total
## Batch01 1 1 1 1 1 1 1 7
## Batch02 1 1 1 1 1 1 1 7
## Batch03 1 1 1 1 1 1 1 7
## Batch04 1 0 1 0 1 0 1 4
## Batch05 1 0 1 0 1 0 1 4
## Batch06 1 0 1 0 1 0 1 4
## Batch07 1 1 1 1 1 1 1 7
## Batch08 1 1 1 1 1 1 1 7
## Batch09 1 1 1 1 1 1 1 7
## Batch10 1 0 1 0 1 0 0 3
## Batch11 1 0 1 0 1 0 0 3
## Batch12 1 0 1 0 1 0 0 3
## Batch13 1 1 1 1 1 1 1 7
## Batch14 1 1 1 1 1 1 1 7
## Batch15 1 1 1 1 1 1 1 7
## Batch16 1 0 1 0 1 0 0 3
## Batch17 1 0 1 0 1 0 0 3
## Batch18 1 0 1 0 1 0 0 3
## Batch19 1 1 1 1 1 1 1 7
## Batch20 1 1 1 1 1 1 1 7
## Batch21 1 1 1 1 1 1 1 7
## Batch22 1 0 1 0 1 0 0 3
## Batch23 1 0 1 0 1 0 0 3
## Batch24 1 0 1 0 1 0 0 3
## 24 12 24 12 24 12 15 123

write.csv(Dist,"Table/Dist.csv")

This table presented status of the long-term stability data under real-time real conditions in normal cold chain (2 to 8ºC) collected at different time points (months) for 24 months storage.

## Distribution of ECTC stability data time points by batch

ECTC_table <- table(ECTC$batch,ECTC$emonth)
ECTC_table_1 <- cbind(ECTC_table,Total=margin.table(ECTC_table, 1))
ECTC_table_2 <- cbind(t(margin.table(ECTC_table, 2)) ,Total=sum(margin.table(ECTC_table, 1)))
ECTC_Dist <- rbind(ECTC_table_1,Total = ECTC_table_2)

ECTC_Dist

## 0 6 12 24 Total
## Batch04 6 6 6 6 24
## Batch05 6 6 6 6 24
## Batch06 6 6 6 6 24
## Batch10 6 6 6 0 18
## Batch11 6 6 6 0 18
## Batch12 6 6 6 0 18
## Batch16 6 6 6 0 18
## Batch17 6 6 6 0 18
## Batch18 6 6 6 0 18
## Batch22 6 6 6 0 18
## Batch23 6 6 6 0 18
## Batch24 6 6 6 0 18
## 72 72 72 18 234

write.csv(ECTC_Dist,"Table/ECTC_Dist.csv")

This table presented status of stability data under accelerated storage conditions (40ºC) collected at different time points (days) at 0, 3, 7, 10, 12, 14 days. There were 4 different exposed months: very early storage period at 0 and 6 months, medium storage period at 12 months, and very late storage period at 24 months.

## Descriptive statistics of real-time stability data (antigen contents) by data time point

The antigen contents as stability indicating parameter was descriptively summarized and graphically explored by time points.

RT_antigen <- ddply(RT, .(nmonth), summarize,
 Nodata=length(nmonth),
 mantigen = mean(antigen),
 sdantigen = sd(antigen))

RT_BasicStat <- data.frame(ObsTimePoint = RT_antigen$nmonth,
 Nodata=RT_antigen$Nodata,
 antigen=paste(as.character(round(RT_antigen$mantigen,2)),"±",as.character(round(RT_antigen$sdantigen,2))))

RT_BasicStat

## ObsTimePoint Nodata antigen
## 1 0 12 924.12 ± 23.55
## 2 3 12 908.88 ± 23.5
## 3 6 12 894.6 ± 23.73
## 4 9 12 873.82 ± 27.73
## 5 12 12 869.13 ± 22.96
## 6 18 12 859.29 ± 19.16
## 7 24 12 845.01 ± 13.11

write.csv(RT_BasicStat,"Table/RT_BasicStat.csv")

This table descriptively presented the mean and standard deviation (SD) of observed antigen contents under real-time real condition, which were above acceptable criteria (800LEU) at each time point for 24 months.

## Plot of antigen contents of real-time stability data over time

We will see the antigen mean plot with error bar by time using ggplot() with geon_errorbar().

ggplot(RT_antigen, aes(x=nmonth, y=mantigen)) +
 geom_point(aes(color = "Mean"), shape = 21, size = 3) +
 geom_errorbar(aes(ymin=mantigen-sdantigen, ymax=mantigen+sdantigen,color = "Mean \u00B1 SD"), width = 0.2) +
 scale_color_manual(name = "Legend", values = c("#ff0000", "#ff0000")) +
 guides(colour = guide_legend(override.aes = list(linetype = c("blank", "solid"), shape = c(21, NA)))) +
 geom_line(colour = "red") +
 scale_x_continuous(breaks=c(0,3,6,9,12,18,24)) +
 scale_y_continuous(breaks=c(750,800,850,900,950,1000),limits=c(750, 1000)) +
 ggtitle("Antigen contents at 2 to 8°C") +
 xlab("Time (Months)") +
 ylab("Antigen contents") +
 geom_hline(yintercept=800, linetype="dashed", colour = "red") +
 geom_text(x=3, y=810, label="Reference line, LL=800LEU") +
 theme_classic() +
 theme(plot.title = element_text(size=14, face="bold",hjust = 0.5),
 legend.title = element_blank(),
 legend.position="bottom",
 legend.background = element_rect(size=0.5, linetype="solid",colour ="gray"),
 panel.border = element_rect(colour = "black", fill = NA))


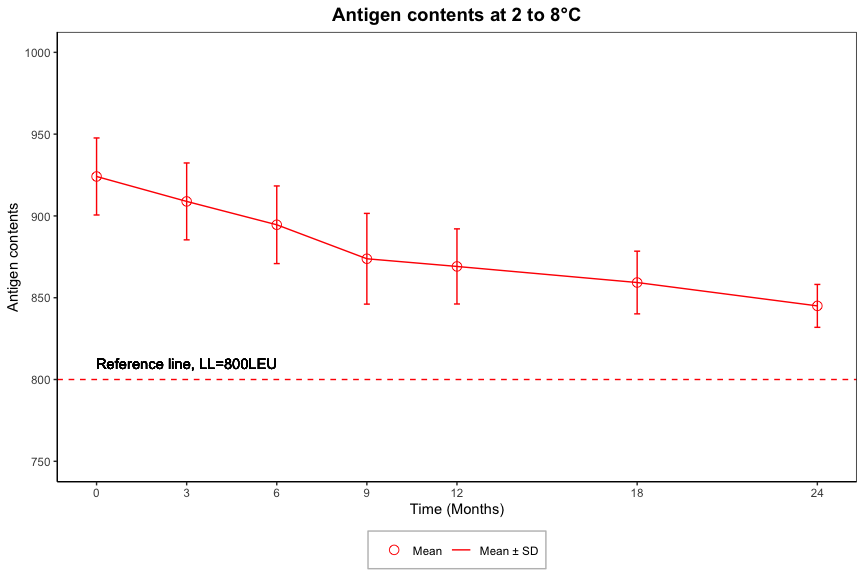


ggsave("Figure/FigureRT.pdf", units = "in")

## Descriptive statistics of ECTC stability data (antigen content) by data time point

ECTC_antigen <- ddply(ECTC, .(ndays), summarize,
 Nodata=length(ndays),
 mantigen = mean(antigen), sdantigen = sd(antigen))

ECTC_BasicStat <- data.frame(ObsTimePoint = ECTC_antigen$ndays,
 Nodata=ECTC_antigen$Nodata,
 antigen=paste(as.character(round(ECTC_antigen$mantigen,2)),"±",as.character(round(ECTC_antigen$sdantigen,2))))

ECTC_BasicStat

## ObsTimePoint Nodata antigen
## 1 0 39 904.04 ± 18.03
## 2 3 39 892.63 ± 22.01
## 3 7 39 876.02 ± 20.23
## 4 10 39 875.8 ± 20.85
## 5 12 39 865.12 ± 25.79
## 6 14 39 850.88 ± 23.37

write.csv(ECTC_BasicStat,"Table/ECTC_BasicStat.csv")

This table descriptively presented the mean and standard deviation (SD) of observed antigen contents under ECTC, which were above acceptable criteria (800LEU) at each time point for 14 days.

## Plot of antigen contents of ECTC stability data over time

ggplot(ECTC_antigen, aes(x=ndays, y=mantigen)) +
 geom_point(aes(color = "Mean"), shape = 21, size = 3) +
 geom_errorbar(aes(ymin=mantigen-sdantigen, ymax=mantigen+sdantigen,color = "Mean \u00B1 SD"), width = 0.2) +
 scale_color_manual(name = "Legend", values = c("#ff0000", "#ff0000")) +
 guides(colour = guide_legend(override.aes = list(linetype = c("blank", "solid"), shape = c(21, NA)))) +
 geom_line(colour = "red") +
 scale_x_continuous(breaks=c(0,3,7,10,12,14)) +
 scale_y_continuous(breaks=c(750,800,850,900,950,1000),limits=c(750, 1000)) +
 ggtitle("Antigen contents at 40°C") +
 xlab("Time (Days)") +
 ylab("Antigen contents") +
 geom_hline(yintercept=800, linetype="dashed", colour = "red") +
 geom_text(x=3, y=810, label="Reference line, LL=800LEU") +
 theme_classic() +
 theme(plot.title = element_text(size=14, face="bold",hjust = 0.5),
 legend.title = element_blank(),
 legend.position="bottom",
 legend.background = element_rect(size=0.5, linetype="solid",colour ="gray"),
 panel.border = element_rect(colour = "black", fill = NA))


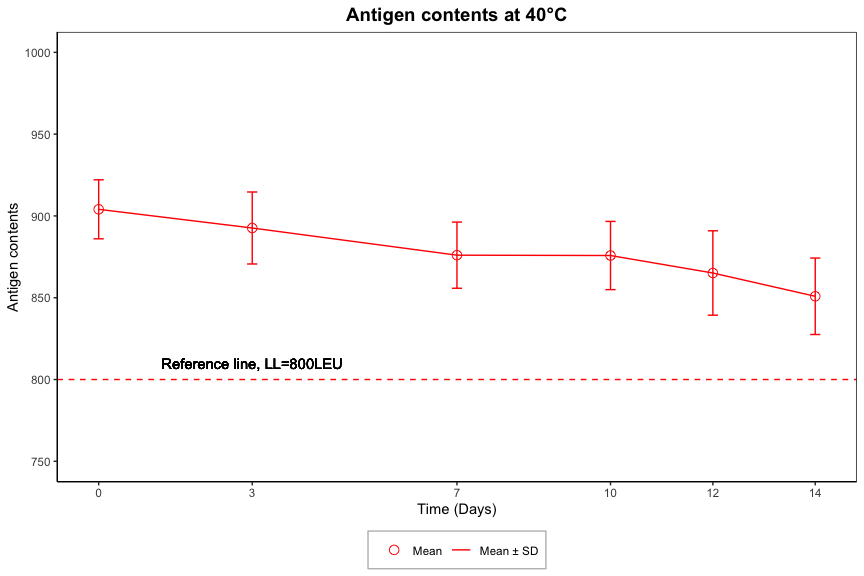


ggsave("Figure/FigureECTC.pdf", units = "in")

# Consideration of statistical models

## Building test models

Linear regression model using lm() will be used for diagnosis. We set a model that a time (months/days) and batch has effect on antigen contents. Here we will show the diagnosis results using model_RT and model_ECTC.

model_RT <- lm(log(antigen) ~ nmonth + factor(batch), data=RT)

model_ECTC <- lm(log(antigen) ~ ndays + factor(batch), data=ECTC)

## Nonlinearity

### Component+Residual (Partial Residual) Plots

You should see a smoothing line that models the residuals of your predictor against your dependent variable (i.e., the loess line). The dashed line represents the line of best fit. If your smoothing line seems to be similarly linear as your dashed line, you’re good. If the smoothing line appears curved relative to the dashed line, you likely have a linearity problem. Here you can control the degree of smoothing. Default value is span = 0.75.

# crPlots with span=0.75 of model of log(antigen) at RT (2 to 8°C)
crPlots(model_RT, smooth=list(span=0.75))


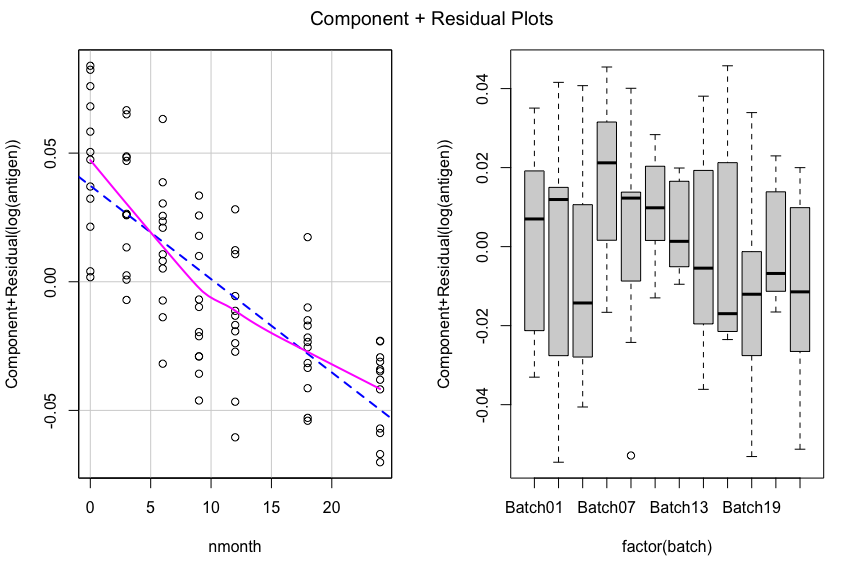


# crPlots with span=0.95 of model of log(antigen) at ECTC (40°C)
crPlots(model_ECTC , smooth=list(span=0.95))


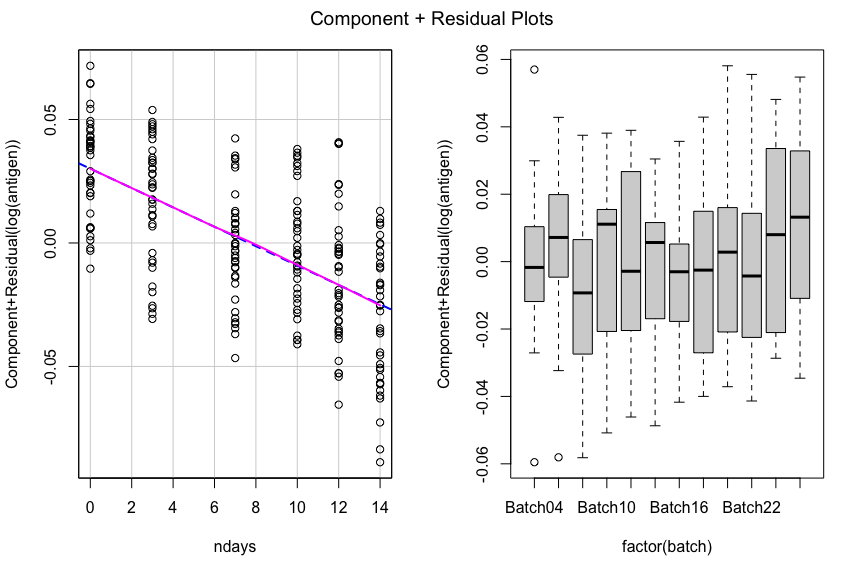


pdf("Figure/ResidualPlot.pdf")
crPlots(model_RT, smooth=list(span=0.75))
crPlots(model_ECTC , smooth=list(span=0.95))
dev.off()

## quartz_off_screen
## 2

## Unusual and influence data

### Standard diagnostic plots

**olsrr** offers the following tools to detect influence data with threshold

- Studentized Residual Plot
- Cook’s D Bar Plot
- Studentized Residuals vs Leverage Plot
- DFFITs Plot

# Standard diagnostic plots of model of log(antigen) at RT (2 to 8°C)
ols_plot_resid_stud(model_RT)


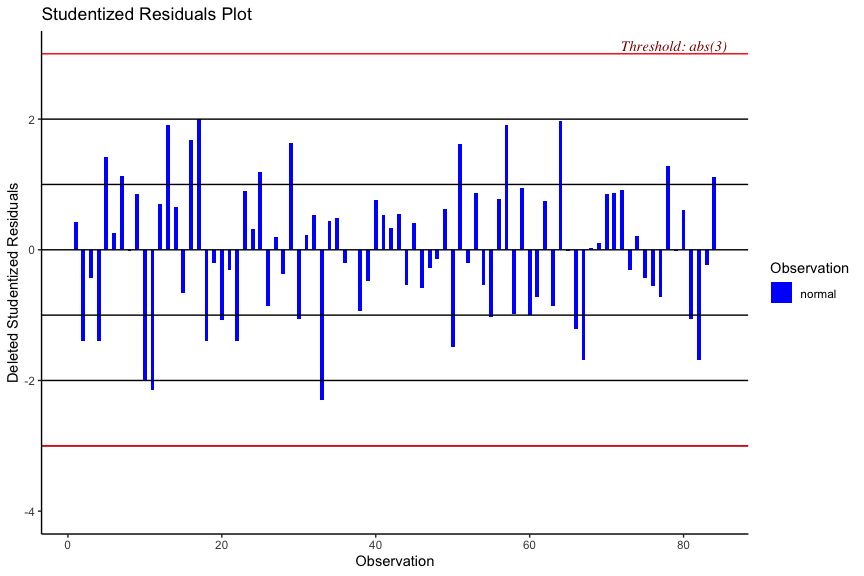


ols_plot_cooksd_bar(model_RT)


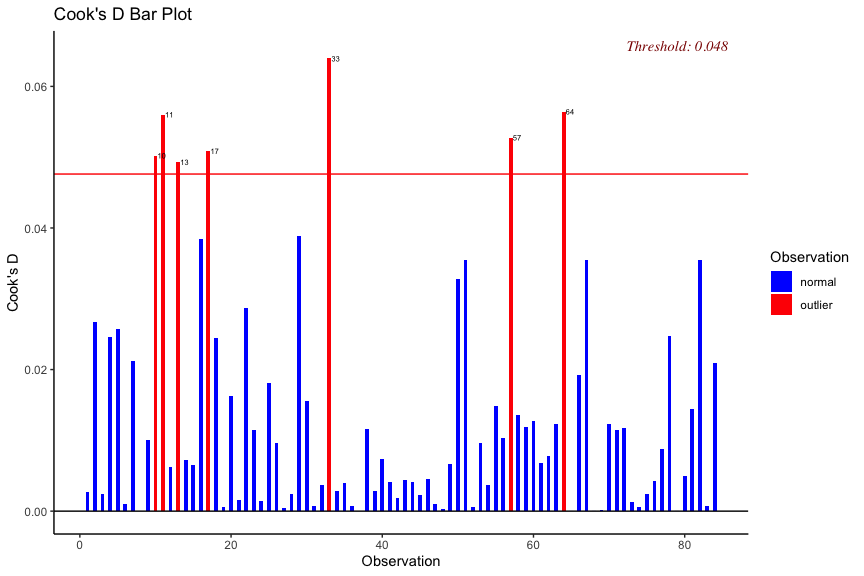


ols_plot_resid_lev(model_RT)


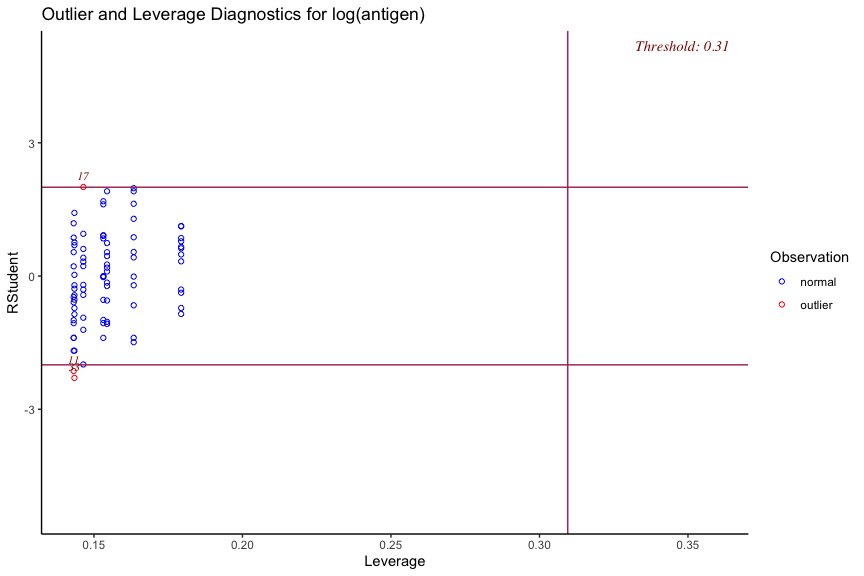


ols_plot_dffits(model_RT)


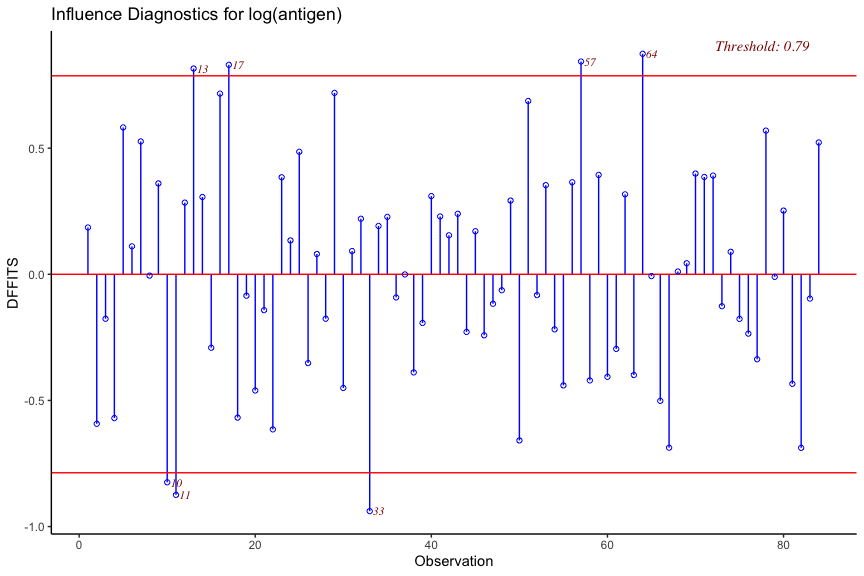


# Standard diagnostic plots of model of log(antigen) at ECTC (40°C)
ols_plot_resid_stud(model_ECTC)


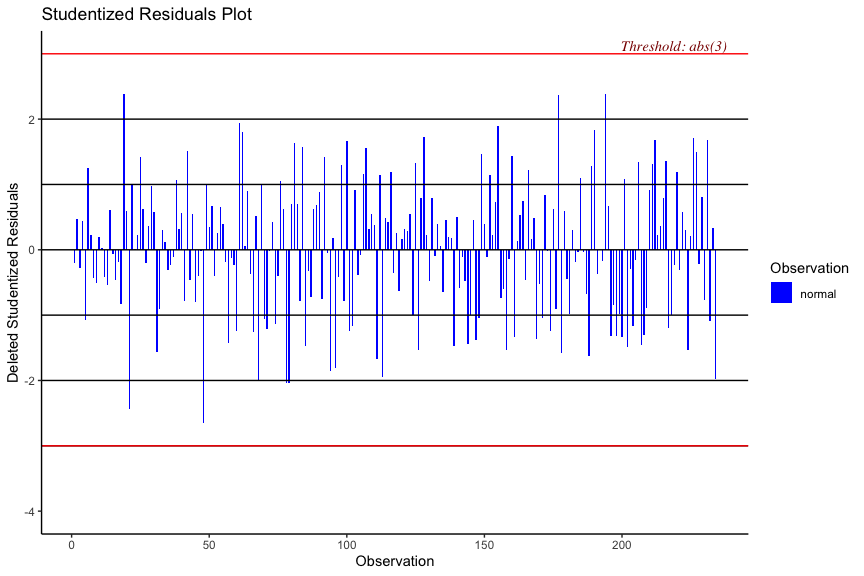


ols_plot_cooksd_bar(model_ECTC)


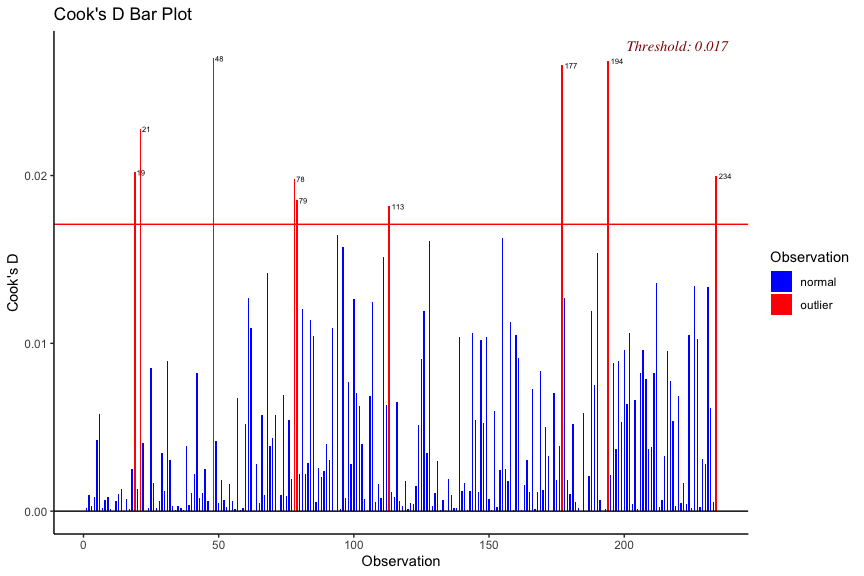


ols_plot_resid_lev(model_ECTC)


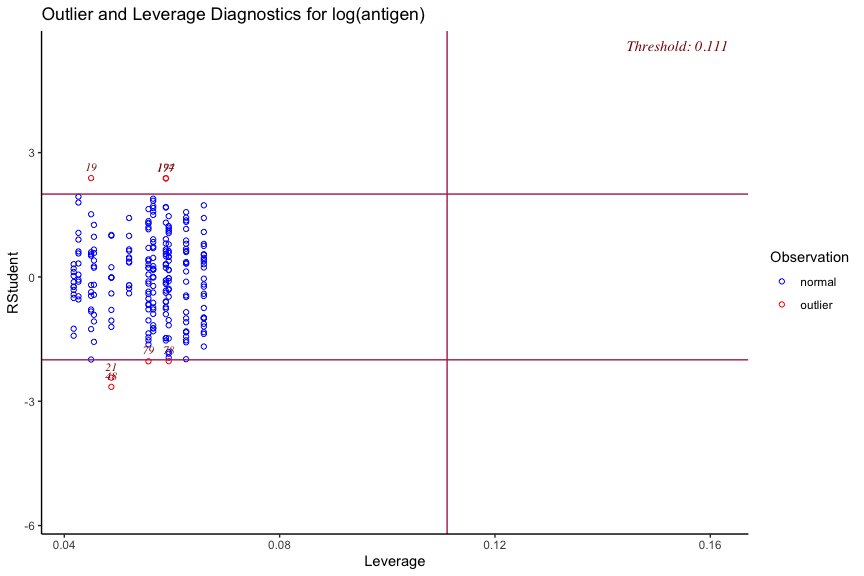


ols_plot_dffits(model_ECTC)


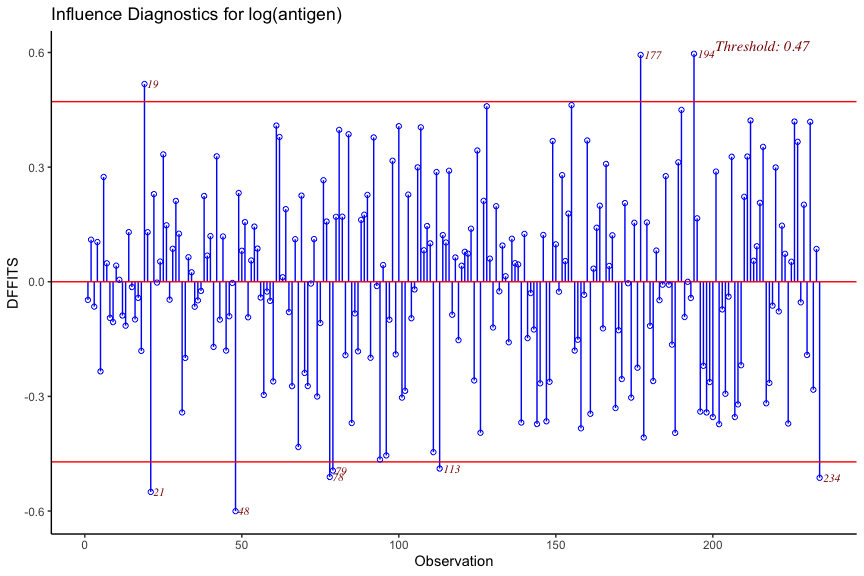


pdf("Figure/CookPlot_RT_antigen.pdf")
ols_plot_resid_stud(model_RT)
ols_plot_cooksd_bar(model_RT)
ols_plot_resid_lev(model_RT)
ols_plot_dffits(model_RT)

ols_plot_resid_stud(model_ECTC)
ols_plot_cooksd_bar(model_ECTC)
ols_plot_resid_lev(model_ECTC)
ols_plot_dffits(model_ECTC)
dev.off()

## quartz_off_screen
## 2

## Normality of residuals

### Assess normality of residuals using Shapiro-Wilk W test

The null hypothesis of these tests is that “sample distribution is normal”. If the test is significant, the distribution is non-normal.

# Shapiro-Wilk W test of model of log(antigen) at RT (2 to 8°C)
shapiro_test(model_RT$residuals)

## # A tibble: 1 x 3
## variable statistic p.value
## <chr> <dbl> <dbl>
## 1 model_RT$residuals 0.987 0.547

# Shapiro-Wilk W test of model of log(antigen) at ECTC (40°C)
shapiro_test(model_ECTC$residuals)

## # A tibble: 1 x 3
## variable statistic p.value
## <chr> <dbl> <dbl>
## 1 model_ECTC$residuals 0.993 0.325

## Heteroscedasticity

### Breusch-Pagan test

Breusch Pagan Test was introduced by Trevor Breusch and Adrian Pagan in 1979. It is used to test for heteroskedasticity in a linear regression model. It test whether variance of errors from a regression is dependent on the values of a independent variable.

Null Hypothesis: Equal/constant variances.

Alternative Hypothesis: Unequal/non-constant variances.

# Breusch-Pagan test of model of log(antigen) at RT (2 to 8°C)
bptest(model_RT)

##
## studentized Breusch-Pagan test
##
## data: model_RT
## BP = 18.418, df = 12, p-value = 0.1036

# Breusch-Pagan test of model of log(antigen) at ECTC (40°C)
bptest(model_ECTC)

##
## studentized Breusch-Pagan test
##
## data: model_ECTC
## BP = 10.71, df = 12, p-value = 0.5539

### White’s test

We can use dptest() for White Test though subtle distinctions play a crucial role here.

# White's test of model of log(antigen) at RT (2 to 8°C)
RT$nbatch <- as.numeric((factor(RT$batch))) #Convert Factor to Numeric
bptest(model_RT, ~ nmonth + nbatch + I(nmonth^2) + I(nbatch^2) + I(nmonth*nbatch), data=RT)

##
## studentized Breusch-Pagan test
##
## data: model_RT
## BP = 7.5563, df = 5, p-value = 0.1824

# White's test of model of log(antigen) at ECTC (40°C)
ECTC$nbatch <- as.numeric((factor(ECTC$batch)))
bptest(model_ECTC, ~ nmonth + nbatch + I(nmonth^2) + I(nbatch^2) + I(nmonth*nbatch) , data=ECTC)

##
## studentized Breusch-Pagan test
##
## data: model_ECTC
## BP = 6.2336, df = 5, p-value = 0.2841

### Residuals vs Fitted Plots

# Residuals vs Fitted Plots of model of log(antigen) at RT (2 to 8°C)
plot(model_RT, which=1)


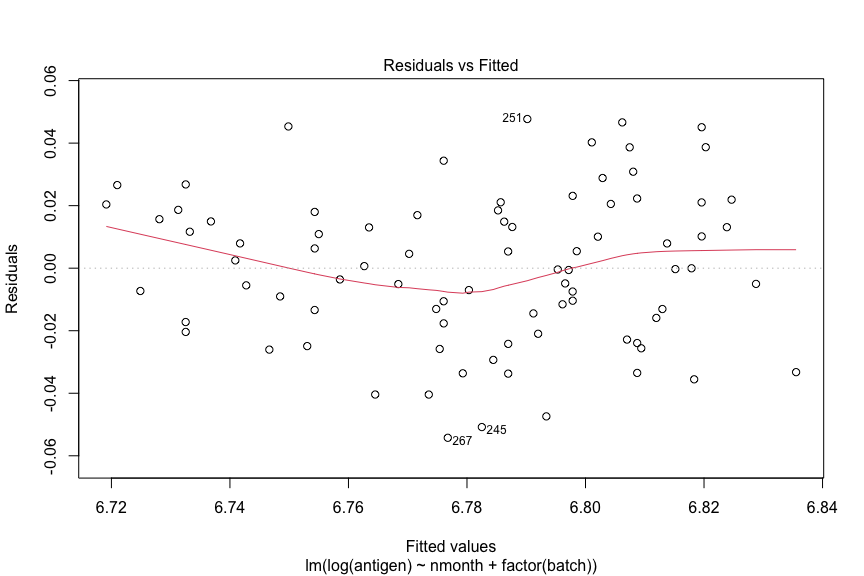


# Residuals vs Fitted Plots of model of log(antigen) at ECTC (40°C)
plot(model_ECTC, which=1)


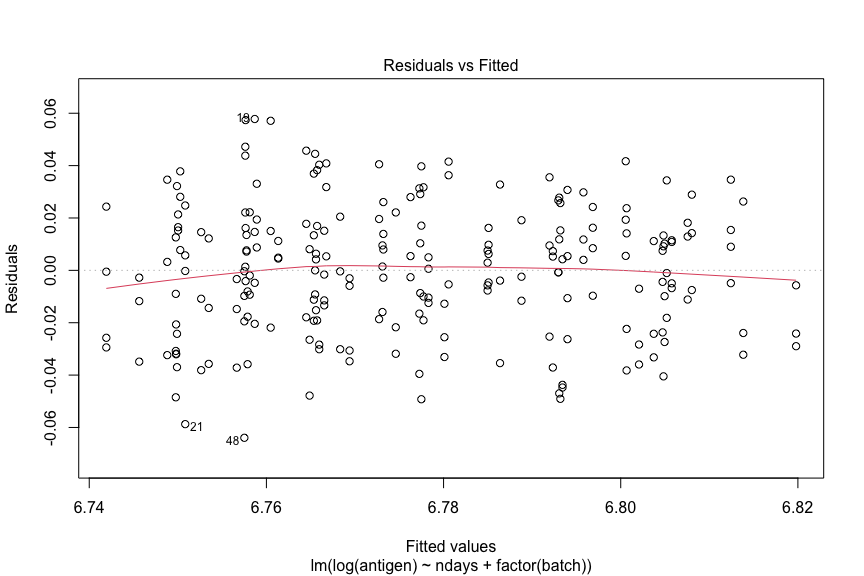


To assess the homoscedasticity assumption, please make sure that the residuals are equally spread around the y = 0 line.

pdf("Figure/StandardizedResidualPlot_ECTC_antigen.pdf")
 plot(model_RT, which=1)
 plot(model_ECTC, which=1)
dev.off()

## quartz_off_screen
## 2

## Independence

### Durbin-Watson Test

dwtest() present a simple test to determine whether there is autocorrelation (aka serial correlation), i.e. where there is a (linear) correlation between the error term for one observation and the next.

Null hypothesis: There is no correlation among the residuals.

Alternative hypothesis: The residuals are autocorrelated.

# Durbin-Watson Test of model of log(antigen) at RT (2 to 8°C)
dwtest(model_RT)

##
## Durbin-Watson test
##
## data: model_RT
## DW = 2.1704, p-value = 0.3106
## alternative hypothesis: true autocorrelation is greater than 0

# Durbin-Watson Test of model of log(antigen) at ECTC (40°C)
dwtest(model_ECTC)

##
## Durbin-Watson test
##
## data: model_ECTC
## DW = 2.2023, p-value = 0.7821
## alternative hypothesis: true autocorrelation is greater than 0

## Poolability

The stability data from different combinations of factors should not be combined unless supported by statistical tests for poolability. Analysis of covariance can be employed to test the difference in slopes and intercepts of the regression lines among factors and factor combinations.

# Models of log(antigen) at RT (2 to 8°C)
 Full <- lm(log(antigen) ~ nmonth + factor(batch) + nmonth:factor(batch), data=RT)
 NonInt <- lm(log(antigen) ~ nmonth + factor(batch) , data=RT)
 NonFac <- lm(log(antigen) ~ nmonth + nmonth:factor(batch), data=RT)

 ## Slope test
 anova(NonInt, Full)

## Analysis of Variance Table
##
## Model 1: log(antigen) ~ nmonth + factor(batch)
## Model 2: log(antigen) ~ nmonth + factor(batch) + nmonth:factor(batch)
## Res.Df RSS Df Sum of Sq F Pr(>F)
## 1 71 0.049112
## 2 60 0.043620 11 0.0054922 0.6868 0.7456

## Intercepts test
 anova(NonFac, Full)

## Analysis of Variance Table
##
## Model 1: log(antigen) ~ nmonth + nmonth:factor(batch)
## Model 2: log(antigen) ~ nmonth + factor(batch) + nmonth:factor(batch)
## Res.Df RSS Df Sum of Sq F Pr(>F)
## 1 71 0.048247
## 2 60 0.043620 11 0.0046269 0.5786 0.8387

# Models of log(antigen) at ECTC (40°C)
 Full <- lm(log(antigen) ~ nmonth + factor(batch) + nmonth:factor(batch), data=ECTC)
 NonInt <- lm(log(antigen) ~ nmonth + factor(batch) , data=ECTC)
 NonFac <- lm(log(antigen) ~ nmonth + nmonth:factor(batch), data=ECTC)

 ## Slope test
 anova(NonInt, Full)

## Analysis of Variance Table
##
## Model 1: log(antigen) ~ nmonth + factor(batch)
## Model 2: log(antigen) ~ nmonth + factor(batch) + nmonth:factor(batch)
## Res.Df RSS Df Sum of Sq F Pr(>F)
## 1 221 0.13849
## 2 210 0.12921 11 0.0092767 1.3707 0.1886

## Intercepts test
 anova(NonFac, Full)

## Analysis of Variance Table
##
## Model 1: log(antigen) ~ nmonth + nmonth:factor(batch)
## Model 2: log(antigen) ~ nmonth + factor(batch) + nmonth:factor(batch)
## Res.Df RSS Df Sum of Sq F Pr(>F)
## 1 221 0.13496
## 2 210 0.12921 11 0.0057532 0.85 0.5903

According to the following criteria (Ref. ICH Q1E. Evaluation for Stability Data. B3.2. Tests for Poolability), test of equality of slope and test of intercept will determine if the combined data will be used in the model.

| Equality of Slope p-value | Equality of Intercept p-value | Slope | Intercept | Description |
| --- | --- | --- | --- | --- |
| ≤ 0.25 | NA | Separate | Separate | Not Parallel |
| > 0.25 | ≤ 0.25 | Common | Separate | Parallel |
| > 0.25 | > 0.25 | Common | Common | One Line |

The Poolability test result using general linear model and criteria (ICH Q1E. Evaluation for Stability Data. B3.2. Tests for Poolability) was suggested separate intercept and separate slope. Therefore, individual mean regression line and 95% confidence interval by batch was calculated from the linear regression model.

# Evaluation of vaccines for use under real-time, real-condition in normal cold chain

## Plot of antigen contents under real-time, real-condition over time

glance_RT <- glance(model_RT)
augment_RT <- augment(model_RT)
mf_RT <- merge(augment_RT,glance_RT)
mf_RT$LCLM <- mf_RT$.fitted - qt(.975, mf_RT$df.residual) * sqrt((mf_RT$.hat) * mf_RT$sigma^2)
mf_RT$UCLM <- mf_RT$.fitted + qt(.975, mf_RT$df.residual) * sqrt((mf_RT$.hat) * mf_RT$sigma^2)

lm_RT <- lm(log(antigen) ~ nmonth, data=RT)

ggplot(mf_RT, aes(x=nmonth, y=`log(antigen)`, color=`factor(batch)`)) +
 geom_point() +
 geom_line(aes(x=nmonth, y=.fitted), linetype="dashed") +
 geom_ribbon(aes(x=nmonth, ymin = LCLM, ymax = UCLM, fill=`factor(batch)`), alpha=0.05, lty=0) +
 geom_text(x=20, y=6.65, label="Dashed line: Predicted Fit", col="black", size=5) +
 geom_abline(slope = coef(lm_RT)[[2]], intercept = coef(lm_RT)[[1]], col="blue") +
 geom_text(x=15, y=6.90, label="Blue line: Predicted line using common slope and intercept", col="blue", size=5) +
 scale_x_continuous(breaks=c(0,3,6,9,12,18,24)) +
 scale_y_continuous(breaks=c(6.6, 6.7, 6.8, 6.9, 7.0, 7.1, 7.2),limits=c(6.65, 6.95)) +
 ggtitle("log-transformed antigen contents at 2 to 8°C") +
 xlab("Time (Months)") +
 ylab("log-transformed antigen contents") +
 geom_hline(yintercept=6.68, linetype="dashed", color = "red") +
 geom_text(x=5, y=6.7, label="Reference line, 6.68 = log(LL=800LEU)", col="red", size=5) +
 theme_classic() +
 theme(plot.title = element_text(hjust = 0.5),legend.position="bottom", legend.title=element_blank())


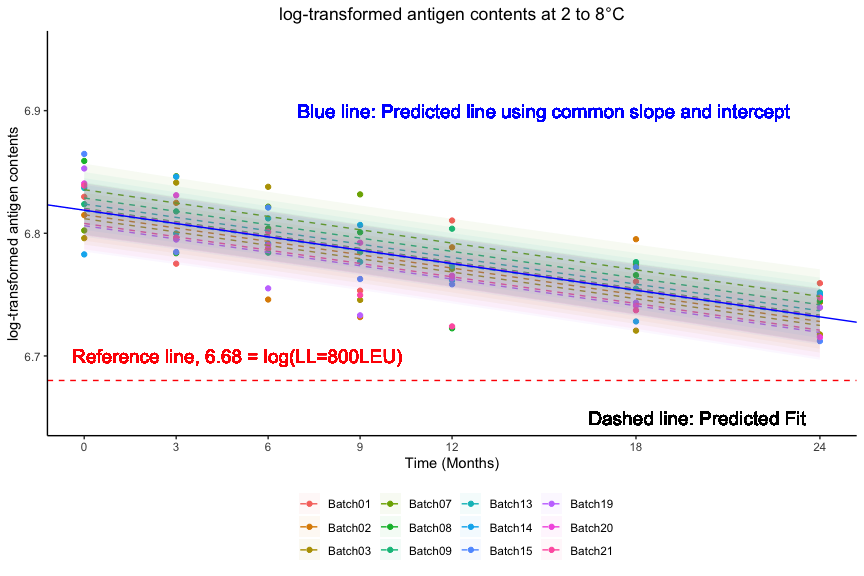


ggsave("Figure/FigureModelRT.pdf", units = "in")

# Stability evaluation of vaccines for use under ECTC

## Plot of antigen contents under ECTC over time

glance_ECTC <- glance(model_ECTC)
augment_ECTC <- augment(model_ECTC)
mf_ECTC <- merge(augment_ECTC,glance_ECTC)
mf_ECTC$LCLM <- mf_ECTC$.fitted - qt(.975, mf_ECTC$df.residual) * sqrt((mf_ECTC$.hat) * mf_ECTC$sigma^2)
mf_ECTC$UCLM <- mf_ECTC$.fitted + qt(.975, mf_ECTC$df.residual) * sqrt((mf_ECTC$.hat) * mf_ECTC$sigma^2)

lm_ECTC <- lm(log(antigen) ~ ndays, data=ECTC)

ggplot(mf_ECTC, aes(x=ndays, y=`log(antigen)`, color=`factor(batch)`)) +
 geom_point() +
 geom_line(aes(x=ndays, y=.fitted), linetype="dashed") +
 geom_ribbon(aes(x=ndays, ymin = LCLM, ymax = UCLM, fill=`factor(batch)`), alpha=0.05, lty=0) +
 geom_text(x=12, y=6.65, label="Dashed line: Predicted Fit", col="black", size=5) +
 geom_abline(slope = coef(lm_ECTC)[[2]], intercept = coef(lm_ECTC)[[1]], col="blue") +
 geom_text(x=9, y=6.90, label="Blue line: Predicted line using common slope and intercept", col="blue", size=5) +
 scale_x_continuous(breaks=c(0,3,7,10,12,14)) +
 scale_y_continuous(breaks=c(6.6, 6.7, 6.8, 6.9, 7.0, 7.1, 7.2),limits=c(6.65, 6.95)) +
 ggtitle("log-transformed antigen contents at 40°C") +
 xlab("Time (Days)") +
 ylab("log-transformed antigen contents") +
 geom_hline(yintercept=6.68, linetype="dashed", color = "red") +
 geom_text(x=3, y=6.7, label="Reference line, 6.68 = log(LL=800LEU)", col="red", size=5) +
 theme_classic() +
 theme(plot.title = element_text(hjust = 0.5),legend.position="bottom", legend.title=element_blank())


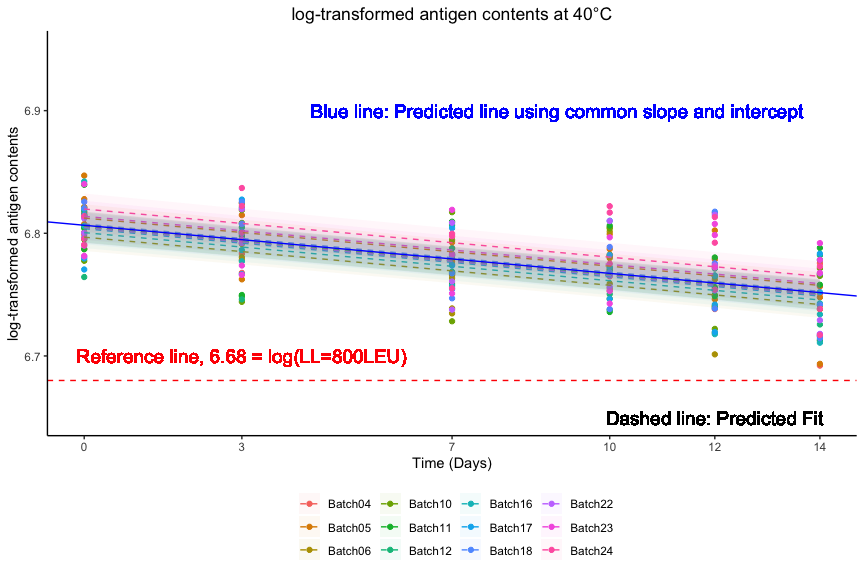


ggsave("Figure/FigureModelECTC.pdf", units = "in")

# ECTC Exposure time and MRP calculation

**Creat sub data set**

### 24 months self-life
ob_antigen_RT_All <- RT

### Divide ECTC data by exposed month
ob_antigen_ECTC_All <- ECTC
ob_antigen_ECTC_00m <- subset(ECTC, emonth == '0')
ob_antigen_ECTC_06m <- subset(ECTC, emonth == '6')
ob_antigen_ECTC_12m <- subset(ECTC, emonth == '12')
ob_antigen_ECTC_24m <- subset(ECTC, emonth == '24')

## Summary of statistical analysis of antigen contents at 2-8°C

### Uncertainty function

Uncertainty <- function(data1, model, Batch, Dataset, months)
{
 glmX <- glm(model, data=data1)

 bX <- coef(summary(glmX))[2,1]
 sbX <- coef(summary(glmX))[2,2]
 sX <- sigma(glmX)
 tX <- months
 z <- qnorm(.95)
 uX <- z*sqrt(sX^2 + (tX*sbX)^2)

 Results <- cbind(Batch, Dataset, ShelfLife = tX, slope = round(bX,6), SEslope = round(sbX,6), rSTD = round(sX,4), Uncertainty = round(uX,4))
 return(Results)
}

Results_RT_24m <- Uncertainty(data1=ob_antigen_RT_All, model="log(antigen) ~ nmonth + factor(batch)", Batch ="All 12 batches", Dataset = "0-24M", months = 24)

Table_Uncertainty <- rbind(Results_RT_24m)

Table_Uncertainty

## Batch Dataset ShelfLife slope SEslope rSTD
## [1,] "All 12 batches" "0-24M" "24" "-0.003628" "0.000366" "0.0263"
## Uncertainty
## [1,] "0.0456"

write.csv(Table_Uncertainty,"Table/Table_Uncertainty.csv")

## Summary of statistical analysis of antigen contents at 40°C

### MRP(minimum release potency) calcultion function

MRP <- function( data1, data2, model, Dataset, months, days, LL)
{
 glmX <- glm(model, data=data1)

 bX <- coef(summary(glmX))[2,1]
 sbX <- coef(summary(glmX))[2,2]
 sX <- sigma(glmX)
 tX <- months
 z <- qnorm(.95)
 uX <- z*sqrt(sX^2 + (tX*sbX)^2)

 glmY <- glm(model, data=data2)

 bY <- coef(summary(glmY))[2,1]
 sbY <- coef(summary(glmY))[2,2]
 sY <- sigma(glmY)

 t <- days
 tY <- t/30
 uY <- z*sqrt(sX^2 + (tX*sbX)^2 + (tY*sbY)^2)

 bX <- if(bX > 0) 0 else bX
 d1Y <- tX*bX + tY*bY - uY
 lMRP <- log(LL) - d1Y
 MRP <- exp(lMRP)

 Results <- cbind(Dataset, ShelfLife = tX, Days = t, slope = round(bY,6), SEslope = round(sbY,6), rSTD = round(sY,4), Uncertainty = round(uY,4), MRP = round(MRP,0))
 return(Results)
}

LL <- 800

MRP(data1=ob_antigen_RT_All, data2=ob_antigen_ECTC_All, model="log(antigen) ~ nmonth + factor(batch)", Dataset = "All pooled 12 batches", months = 24, days = 3, LL = LL)

## Dataset ShelfLife Days slope SEslope rSTD
## [1,] "All pooled 12 batches" "24" "3" "-0.11748" "0.009942" "0.025"
## Uncertainty MRP
## [1,] "0.0456" "924"

Table_MRP

## Dataset ShelfLife Days slope SEslope
## [1,] "All pooled 12 batches " "24" "3" "-0.11748" "0.009942"
## [2,] "All pooled 12 batches " "24" "7" "-0.11748" "0.009942"
## [3,] "All pooled 12 batches " "24" "10" "-0.11748" "0.009942"
## [4,] "All pooled 12 batches " "24" "12" "-0.11748" "0.009942"
## [5,] "All pooled 12 batches " "24" "14" "-0.11748" "0.009942"
## [6,] "12 batches exposed at 0 months" "24" "3" "-0.118629" "0.018841"
## [7,] "12 batches exposed at 0 months" "24" "7" "-0.118629" "0.018841"
## [8,] "12 batches exposed at 0 months" "24" "10" "-0.118629" "0.018841"
## [9,] "12 batches exposed at 0 months" "24" "12" "-0.118629" "0.018841"
## [10,] "12 batches exposed at 0 months" "24" "14" "-0.118629" "0.018841"
## [11,] "12 batches exposed at 6 months" "24" "3" "-0.126544" "0.018649"
## [12,] "12 batches exposed at 6 months" "24" "7" "-0.126544" "0.018649"
## [13,] "12 batches exposed at 6 months" "24" "10" "-0.126544" "0.018649"
## [14,] "12 batches exposed at 6 months" "24" "12" "-0.126544" "0.018649"
## [15,] "12 batches exposed at 6 months" "24" "14" "-0.126544" "0.018649"
## [16,] "12 batches exposed at 12 month" "24" "3" "-0.102528" "0.017323"
## [17,] "12 batches exposed at 12 month" "24" "7" "-0.102528" "0.017323"
## [18,] "12 batches exposed at 12 month" "24" "10" "-0.102528" "0.017323"
## [19,] "12 batches exposed at 12 month" "24" "12" "-0.102528" "0.017323"
## [20,] "12 batches exposed at 12 month" "24" "14" "-0.102528" "0.017323"
## [21,] "3 batches exposed at 24 month" "24" "3" "-0.13644" "0.034428"
## [22,] "3 batches exposed at 24 month" "24" "7" "-0.13644" "0.034428"
## [23,] "3 batches exposed at 24 month" "24" "10" "-0.13644" "0.034428"
## [24,] "3 batches exposed at 24 month" "24" "12" "-0.13644" "0.034428"
## [25,] "3 batches exposed at 24 month" "24" "14" "-0.13644" "0.034428"
## rSTD Uncertainty MRP
## [1,] "0.025" "0.0456" "924"
## [2,] "0.025" "0.0458" "939"
## [3,] "0.025" "0.0459" "950"
## [4,] "0.025" "0.0461" "958"
## [5,] "0.025" "0.0462" "966"
## [6,] "0.0263" "0.0457" "925"
## [7,] "0.0263" "0.0462" "940"
## [8,] "0.0263" "0.0468" "951"
## [9,] "0.0263" "0.0473" "959"
## [10,] "0.0263" "0.0479" "968"
## [11,] "0.026" "0.0457" "925"
## [12,] "0.026" "0.0462" "941"
## [13,] "0.026" "0.0467" "954"
## [14,] "0.026" "0.0472" "962"
## [15,] "0.026" "0.0478" "971"
## [16,] "0.0242" "0.0457" "923"
## [17,] "0.0242" "0.0461" "936"
## [18,] "0.0242" "0.0466" "946"
## [19,] "0.0242" "0.047" "953"
## [20,] "0.0242" "0.0475" "960"
## [21,] "0.024" "0.046" "926"
## [22,] "0.024" "0.0475" "945"
## [23,] "0.024" "0.0494" "960"
## [24,] "0.024" "0.0509" "970"
## [25,] "0.024" "0.0527" "981"

write.csv(Table_MRP,"Table/Table_MRP.csv")
